# Electrochemical Dissolution: Paths in High-Entropy Alloy Composition Space

**DOI:** 10.1007/s44210-025-00057-3

**Published:** 2025-04-29

**Authors:** Mads K. Plenge, Jack K. Pedersen, Luis A. Cipriano, Jan Rossmeisl

**Affiliations:** Department of Chemistry, University of Copenhagen, Universitetsparken 5, 2100 Copenhagen, Denmark

**Keywords:** Electrochemical dissolution, High-entropy alloys, Catalyst stability, Oxygen reduction reaction, Nanoparticle degradation

## Abstract

The stability of a nanoparticle catalyst during electrochemical reaction is crucial for its application. Despite increasing interest in multi-metallic alloy nanoparticles, such as high-entropy alloys (HEAs), for electrocatalysis and emerging models for their catalytic activity, there is limited work on frameworks that can predict the metastability of these alloys under reaction conditions, including stability against electrochemical surface dissolution. Incorporating electrochemical stability in multi-objective optimization would advance HEAs as a catalyst discovery platform. To address the knowledge gap on electrochemical stability, we propose a methodology for simulating the dissolution of n-element alloy nanoparticles comprised of density functional theory and machine-learning regression to calculate the dissolution potentials of the surface atoms. We demonstrate the methodology for the Ag–Au–Cu–Ir–Pd–Pt–Rh–Ru HEA system with the conditions of the oxygen reduction reaction. We investigated trends in stability against dissolution through a compositional grid search for the octo-metallic composition space, uncovering two alloying strategies to increase stability against electrochemical surface dissolution: Alloying with a noble metal or a metal with high relative surface energy. In the simulations, stabilization ensues from forming a protective surface layer, and consequently, the dissolution of persistent alloyed nanoparticles results in core–shell structures. The model enables tracing the evolution of the surface and dissolved composition during electrochemical dissolution, forming paths of dissolution and revealing unretainable surface compositions.

**Graphical Abstract:**

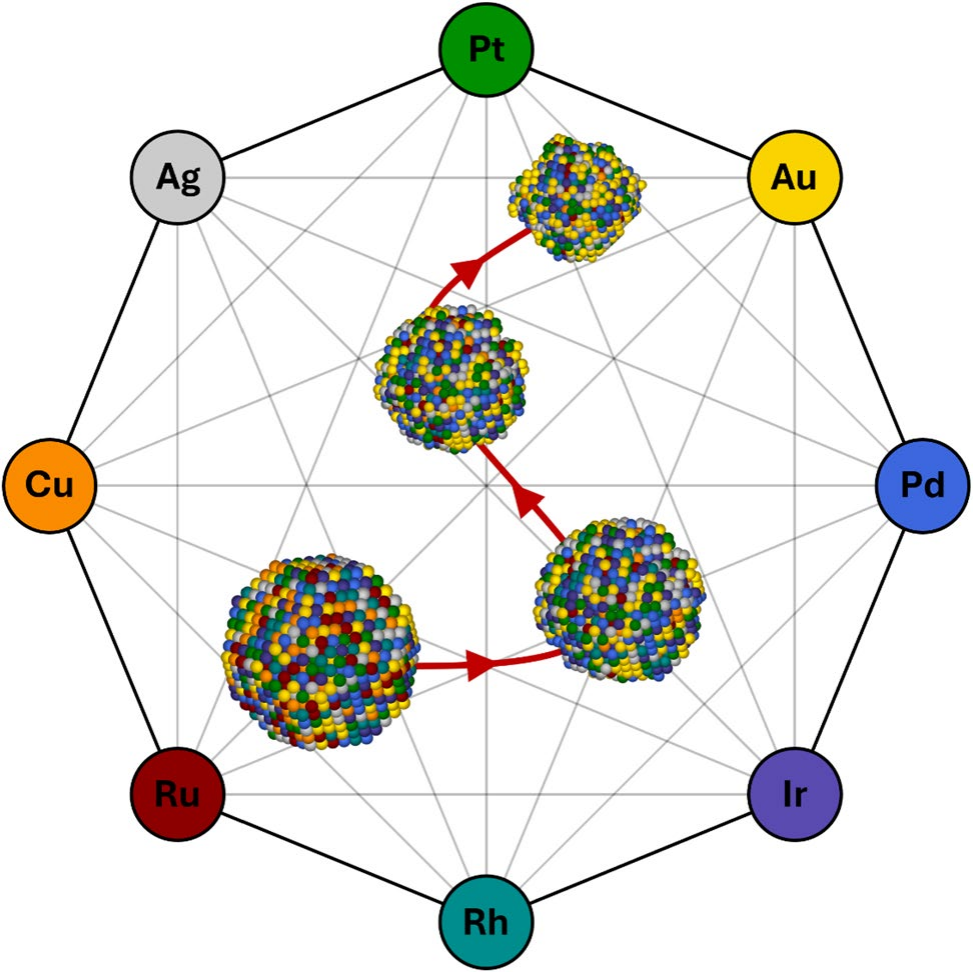

**Supplementary Information:**

The online version contains supplementary material available at 10.1007/s44210-025-00057-3.

## Introduction

Catalysts are set to play an essential role in achieving a full transition to renewable energy sources [1–4]. One key aspect is the electrochemical splitting of water to store renewable energy as green hydrogen and its subsequent use in fuel cells [1, 5–8]. The limiting reaction in hydrogen fuel cells is the cathodic oxygen reduction reaction (ORR), for which Pt/C is the benchmark catalyst [6, 9–11]. Besides Pt not being the theoretical optimal catalyst [9, 12], it is an expensive and scarce element, which hinders large-scale application [7, 10, 11, 13]. Thus, replacing Pt or reducing the amount of Pt, increasing the catalytic activity per Pt atom through alloying, is of great interest [14–16].

Employing nanoparticles as catalysts increases the efficiency in utilizing the catalytically active materials through a high surface-to-volume ratio [14]. A challenging aspect of the ORR is the electrochemical instability due to acidic electrolytes and relatively high operating potentials, causing a significant loss in activity over time due to a decreasing active surface area, which has been studied extensively for Pt and Pt binary alloy nanoparticles [14–22]. The primary attributed mechanisms for the electrochemical degradation of Pt nanoparticles in the ORR environment are Ostwald ripening, agglomeration, particle coalescence, and dissolution of the metal into the electrolyte, for which dissolution has been reported as a predominant degradation mechanism [18, 19, 23–27]. Although significant progress has been made in understanding and modeling electrochemical dissolution and re-deposition, considerable knowledge gaps persist [24, 28–32]. To address these challenges, detailed models of single-metal electrodes have been developed, incorporating the effects of the solvent as well as electron- and ion-transfer mechanisms [33, 34]. Encapsulating all the convoluted degradational effects and mechanisms within one model for multiple alloyed elements is thus a daunting task. This study will, therefore, focus on developing a straightforward approach to modeling the dissolution of alloy nanoparticles to broaden and generalize the understanding of nanoparticle stabilization against dissolution in reaction conditions.

High-entropy alloys (HEAs), consisting of five or more metals randomly distributed in the lattice in near-equal amounts, have gained increasing interest as catalysts [35–40]. Their random configuration of metals provides an array of different adsorption sites, resulting in compositionally tunable distributions of adsorption energies [41–43]. To generalize alloy optimization and to investigate compositional trends enabled by the continuous composition space of HEAs, theoretical work has modeled catalytic activity on HEAs facilitated by machine-learning regression models trained on density functional theory (DFT) calculations of adsorption energies [43–47]. In contrast, as highlighted in a recent review [48], there is currently no theoretical foundation, no applicable descriptors, and only limited experimental data on the electrochemical stability of alloy nanoparticles, including HEAs. A theoretical model, in combination with established activity models, would enable a comprehensive HEA catalyst optimization by multi-objective optimization, fully utilizing HEAs as a catalyst discovery platform. While initial steps toward this approach have been taken [49], a significant potential remains for more advanced modeling of stability under reaction conditions. Preliminary work on a theoretical model for electrochemical stability, conducted by some of the authors [50], simulated the dissolution of mixed Au–Pd nanoparticles by removing surface atoms according to their coordination number (CN). From DFT, they obtained distributions of dissolution potentials for which each metal in different surface structures dissolves. They found that adding a non-corrosive element (Au) in relatively small amounts, e.g., 20 atomic percent (at.%), increases the stability of the Pd nanoparticle against dissolution. In the present study, we expanded on this model by generalizing the dissolution process. To achieve this, we constructed a regression model to calculate the dissolution potential of surface atoms present on multi-metallic nanoparticles within the Ag–Au–Cu–Ir–Pd–Pt–Rh–Ru HEA system.

With the presented methodology, we provide a theoretical framework for uncovering the driving forces and limiting factors of electrochemical stability against the dissolution of multi-metallic nanoparticles. Furthermore, the developed model should be comparable with experiments, *e.g.,* by tracking the dissolved composition of a given multi-metallic system with Inductively Coupled Plasma (ICP) Spectroscopy [51, 52]. With ORR as the demonstrating reaction, stability is quantified to form a composition space for stability by considering the relative amount of (111) surface atoms before and post-dissolution, with the (111) surface assumed to be the more active surface for ORR. Following the work on adsorption energies of HEAs [43, 44], a regression model is trained on DFT simulations and employed to calculate the dissolution potentials of individual surface atoms based on their immediate atomic environment. The regression model uncovers atomistic effects to decrease or increase the surface dissolution potential relative to the bulk dissolution potential. These effects are related to the relative surface energies of the alloyed elements, which, along with each metal’s bulk dissolution potential, i.e., reduction potential, influence stability on a particle scale and the resulting surface composition. Finally, the dissolved structures show surface passivation, resulting in core–shell nanoparticles consistent with the literature, and tracking the changing surface composition reveals paths through composition space.

## Methods

**Fig. 1:**
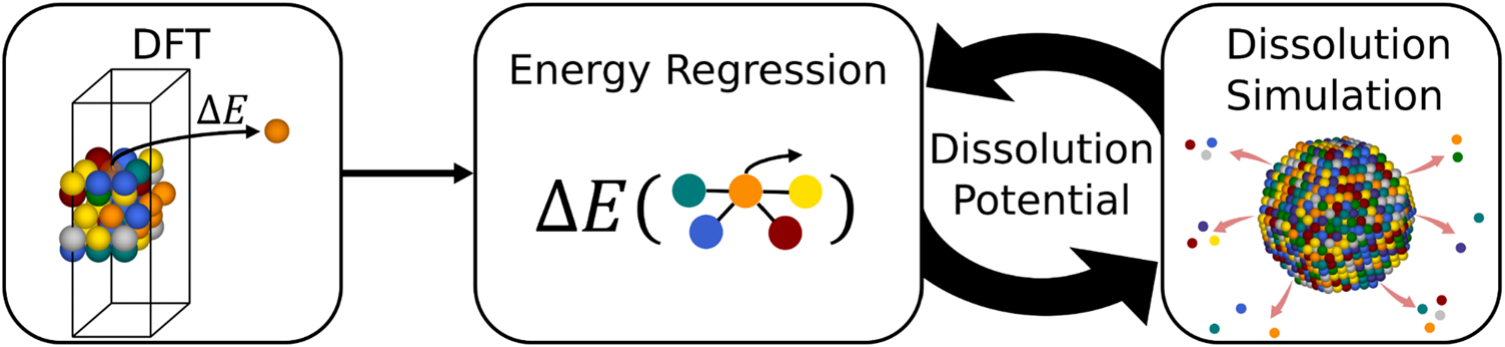
Workflow of the presented methodology, illustrating the use of DFT simulations to calculate change in energy to build a regression model that calculates and updates dissolution potentials during simulation

The workflow for modeling the electrochemical dissolution, illustrated in Fig. 1, entails predicting the dissolution potential of individual surface atoms of a simulated nanoparticle. Combinatorically, an unfathomable number of possible configurations of HEA nanoparticles exist with just a specific size, shape, and composition. Therefore, we propose to consider the surface atoms locally, which enables calculating the energetic cost of dissolving surface atoms from surface slabs of different structures and compositional configurations with DFT, to build a training set for a machine-learning regression model. The final part of the framework is implementing the energy regression model to calculate and update dissolution potentials to simulate dissolution.

### Density Functional Theory Calculations

The energy change ($\Delta E$) associated with removing an atom from a surface structure is determined through DFT calculations using Eq. 1, where ${E}_{\text{slab}}$ and ${E}_{\text{slab}-1}$ are the energies of the structure before and after removing the target atom, respectively. ${E}_{\text{bulk}}$ is the bulk energy of the target atom (listed in Table S1 of the Supplementary Information). The local structure of the surface atom to remove, referred to as the target, is reduced to its immediate coordination environment. Each CN, ranging from 3 to 9, is modeled by a representative structure shown in Fig. S1 and listed in Table S2. The structures model the surfaces present upon initialization of the particle, illustrated in Fig. S2, namely, (111) and (100) facets, edge, and kink (corner) surfaces, in descending CN order from 9 to 6. Lower CNs (3, 4, and 5), occurring as the number of defects from dissolution increases, are modeled by adatoms on the (111), (100), and edge surfaces, respectively.

1 $$\begin{array}{c}\Delta E=\left({E}_{\text{slab}-1}+{E}_{\text{bulk}}\right)-{E}_{\text{slab}} \end{array}$$

A detailed description of the DFT calculations is provided in Section S1 of the Supplementary Information. The calculations cover both single metals and HEA structures. As it is infeasible to calculate all possible configurations within the HEA system with DFT, a subset from 100 uniformly drawn compositions was constructed as training data for a machine-learning regression model. On each surface, the target was substituted with each metal, creating 100 data points of each CN for each metal. A total of 6000 HEA structures were calculated. Relaxed structures deviating from their assigned surfaces and CNs were disregarded from further analysis according to the criteria described in Section S1. An overview of the discarded structures is provided in Table S3.

### Energy Regression Model

Comparing the DFT results for the single metals and HEAs in Figs. S3 and S4 reveals that the HEAs data presents a distribution for each CN for each metal. The constructed model includes the location of the distribution as a parameter, i.e., target metal and CN, while accounting for the neighboring environment as perturbations to capture the distributions. In this initial study, we restrict the regression model to only considering the identity of the neighboring metals surrounding the target atom. A more precise feature is attainable by considering the position of the neighbor atoms and their CNs. However, to make such a model viable in the simulation, several additional structures for each CN that contain more than the current single defect would be needed because, as the particle dissolves, the surfaces will gain more defects and, thereby, the neighboring atoms will have lower CN than the calculated surfaces.

Therefore, a linear approach is adopted to ensure simplicity and interpretability. The linear model employs a multilinear regression for each target metal, one-hot encoding the CNs while encoding the neighboring metals by relative frequency. The predicted change in energy of a target element ($\Delta {E}_{\text{pred}}^{\text{target}}$) is thereby given by Eq. 2, where ${E}_{\text{CN}}^{\text{target}}$ and ${E}_{\text{metal}}^{\text{target}}$ are the learned parameters describing the energy of the CN of the target metal and each neighboring metal’s perturbation to it, respectively. ${N}_{\text{metal}}$ denotes the number of the given metal in the coordinating atoms.

2 $$\begin{array}{*{20}c} {\Delta E_{{{\text{pred}}}}^{{{\text{target}}}} = E_{{{\text{CN}}}}^{{{\text{target}}}} + \frac{1}{{{\text{CN}}}}\sum\limits_{{_{{{\text{metal}} \in {\text{metals}}}} }}^{{}} {E_{{{\text{metal}}}}^{{{\text{target}}}} \cdot N_{{{\text{metal}}}} } } \\ \end{array}$$

**Fig. 2:**
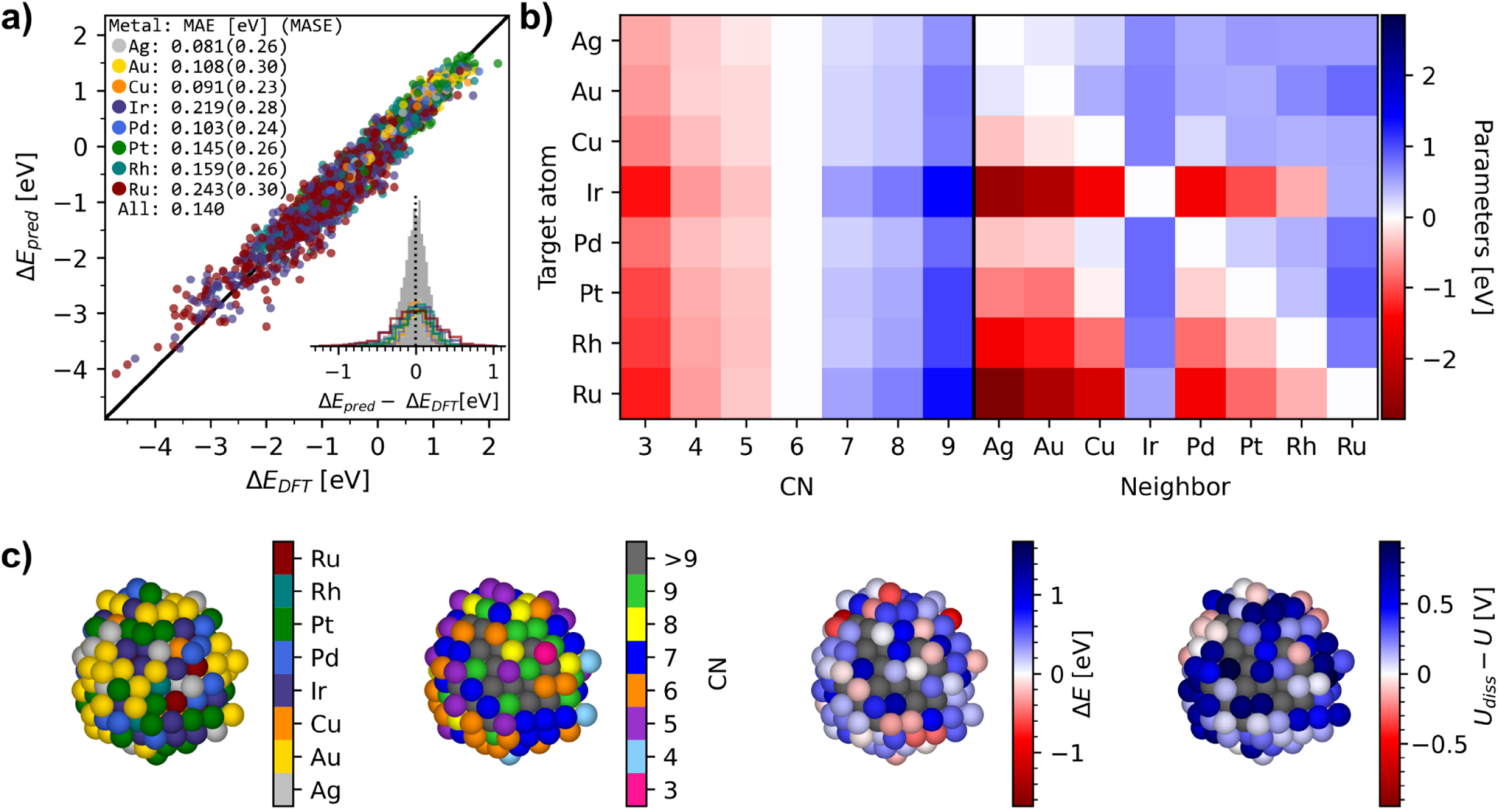
Parity plot from leave-one-out cross-validation of the regression model in **a** and the model parameters in **b**. **c** shows the regression model used on a partly dissolved nanoparticle to calculate dissolution potentials using Eq. 5 (displayed right) from considering the elements, CNs, and $\Delta E$ (shown from left to right)

The regression model was optimized using Sequential Least Squares Programming, in which the parameters for CN = 6 (${\text{E}}_{\text{CN}=6}^{\text{target}}$) and the perturbation of the target metal itself (${E}_{\text{metal}=\text{target}}^{\text{target}}$) were constrained to zero. Thus, the learned parameters are relative to the single metals at CN = 6. The regression model was validated through leave-one-out cross-validation, obtaining a mean absolute error (MAE) of 0.140 eV and root mean square error of 0.193 eV. The performance is illustrated by a parity plot in Fig. 2a. There is a notable difference in accuracy between target metals, primarily caused by difference in ranges of $\Delta E$ values, as indicated by a similar mean absolute scaled error (MASE). The regression model parameters are shown in Fig. 2b and listed in Table S4. In Fig. S5, the neighbor parameters (${E}_{\text{metal}}^{\text{target}}$ in Eq. 2) are plotted against relative surface energies showing a correlation.

### Dissolution Potential

The dissolution potential (${U}_{\text{diss}}$) is given by Eq. 3 [50, 53, 54], where $n$ is the number of transferred electrons in the redox reaction, $e$ is the electron charge, ${U}_{M}$ is the reduction potential of the target metal, and $\Delta E$ is the energy for removing the atom from the surface (Eq. 1).

3 $$\begin{array}{c}{U}_{\text{diss}}=\frac{\Delta E}{ne}+{U}_{M}\end{array}$$

The dissolution potential depends on the metal concentration (${\text{c}}_{\text{M}}$) as given by Eq. 4 [54], where ${U}_{M}^{0}$ is the standard reduction potential of metal M, ${c}^{0}$ is the standard concentration, ${k}_{B}$ is the Boltzmann constant, and $T=298.15$ K is the temperature. pH is considered by the potential via the reversible hydrogen electrode (RHE). The concentration will be assumed to be ${10}^{-6}$ M for all metals, following other studies [19, 20]. Combining Eqs. 3 and 4 while substituting $\Delta E$ for $\Delta {E}_{\text{pred}}^{\text{target}}$ (Eq. 2) provides the estimated dissolution potentials of atoms during simulation in Eq. 5. Table 1 lists the considered reduction potentials and their half-reactions.

**Table 1:** Standard reduction potentials (${\text{U}}_{M}^{0}$) vs. SHE from ref. [55] and reduction potentials at ${10}^{-6}$M

Metal	Half-reaction	$n$	${U}_{M}^{0}$[V]	${U}_{M}({10}^{-6}\text{ M})$[V]
Ag	${\text{Ag}}^{+}+{\text{e}}^{-}\rightleftharpoons \text{Ag}$	1	0.80	0.45
Au	${\text{Au}}^{+}+{\text{e}}^{-}\rightleftharpoons \text{Au}$	1	1.69	1.34
	${\text{Au}}^{3+}+3{\text{e}}^{-}\rightleftharpoons \text{Au}$	3	1.50	1.38
Cu	${\text{Cu}}^{+}+{\text{e}}^{-}\rightleftharpoons \text{Cu}$	1	0.52	0.17
	${\text{Cu}}^{2+}+2{\text{e}}^{-}\rightleftharpoons \text{Cu}$	2	0.34	0.16
Ir	${\text{Ir}}^{3+}+3{\text{e}}^{-}\rightleftharpoons \text{Ir}$	3	1.16	1.04
Pd	${\text{Pd}}^{2+}+2{\text{e}}^{-}\rightleftharpoons \text{Pd}$	2	0.95	0.77
Pt	${\text{Pt}}^{2+}+2{\text{e}}^{-}\rightleftharpoons \text{Pt}$	2	1.18	1.00
Rh	${\text{Rh}}^{+}+{\text{e}}^{-}\rightleftharpoons \text{Rh}$	1	0.60	0.25
	${\text{Rh}}^{3+}+{3\text{e}}^{-}\rightleftharpoons \text{Rh}$	3	0.76	0.64
Ru	${\text{Ru}}^{2+}+2{\text{e}}^{-}\rightleftharpoons \text{Rh}$	2	0.46	0.28

4 $$\begin{array}{c}{U}_{M}\left({\text{c}}_{\text{M}}\right)={U}_{M}^{0}+\frac{{k}_{B}T}{ne} \ln\left(\frac{{c}_{M}}{{\text{c}}^{0}}\right)\end{array}$$

5 $$\begin{array}{c}{U}_{\text{diss}}\approx \frac{\Delta {E}_{\text{pred}}^{\text{target}}}{ne}+{U}_{M}\left({10}^{-6}\text{ M}\right)\end{array}$$

Experimental studies on Pt nanoparticles in ORR conditions have reported that the Pt atoms in small nanoparticles primarily dissolve directly to soluble Pt^2+^ [20, 21, 23], which will be assumed to apply generally in this work. The atoms are assigned the lowest dissolution potential from Eq. 5, considering the different half-reactions of each metal in Table 1. When applicable, the electrolyte should be considered by accounting for the reduction potential of possible complexes formed between the metal and the anion. Figure 2c illustrates an example of applying the regression model on a particle to calculate dissolution potentials, with red indicating soluble atoms on the furthest right particle. The prediction accuracy for the dissolution potentials using the energy regression model, as used in this work, is illustrated by a parity plot in Fig. S6. The MAE of 0.08 V is notably lower than the energy MAE as the energies are scaled by 1/n electrons in Eq. 5. The residuals are more significant for low dissolution potentials. However, in dissolving atoms discretely by dissolution potentials below the applied potential, only the accuracy around the applied potential is relevant. For example, comparing discrete predictions in categorizing target atoms as dissolving versus stable at 0.8 V provides an accuracy of 0.95, as shown in the confusion matrix in Fig. S7.

### Dissolution Simulation

The simulated nanoparticles are generated in ASE [56] as Pt particles with face-centered cubic (fcc) structure using the Wulff construction and by considering the (111) and (100) surfaces, resulting in truncated octahedron nanoparticles that subsequently are populated with elements in a disordered manner while ensuring the desired compositions. The nanoparticle size refers to the distance between two diametrical opposite corner atoms of the initially constructed Pt particle. The simulation proceeds with the following steps:

1. Initiate a particle of a given size, shape, and composition.
2. For all surface atoms: Predict $\Delta E$ and calculate ${U}_{\text{diss}}$ using Eq. 5.
3. Remove surface atoms where ${U}_{\text{diss}}<U$ or CN $<3$.
4. Run particle relaxation scheme.
5. Repeat from 2, until all surface atoms satisfy ${U}_{\text{diss}}\ge U$ or the number of atoms is 0.

Step 4 runs an optional particle relaxation scheme to minimize the total surface energy. To maintain simplicity and increase the number of viable simulations, a straightforward relaxation scheme is employed where atoms move to unoccupied fcc positions to increase coordination. The listed methodology dissolves atoms in batches but could alternatively dissolve atoms one at a time by the lowest dissolution potential. The difference between dissolution and relaxation iteratively versus in batches, including not applying relaxation, is shown in Fig. S8. The batch approach, i.e., influencing several atoms between updates, is chosen for efficiency. In batch relaxation, the repositioning of atoms is prioritized according to improvement in CN, immobilizing atoms affected by atoms with higher priority in the current iteration. Relaxation proceeds until no further achievable improvements.

Analyzing stability across composition space requires applying a quantitative stability measure on the resulting particles. A relevant quantifier for ORR is evaluating concentration changes of the (111) surface by comparing the number of atoms before and after dissolution, mimicking a relative change in the electrochemical active surface area (ECSA), a central parameter in comparing catalytic activity and stability [57, 58]. Additionally, it provides a relatively stable stability parameter as the (111) facet is the most stable surface, and the lower coordinated facets, e.g., (100), are indirectly accounted for by being included in the DFT calculations and the regression model. We denote this stability parameter as ${S}_{d}$ in Eq. 6. The number of atoms (${N}_{111}$) in the (111) surface is estimated by the number of atoms with CN = 9.

6 $$\begin{array}{c}{S}_{d}=\frac{{N}_{111}\left(\text{final}\right)}{{N}_{111}\left(\text{initial}\right)}\end{array}$$

The simulation parameters, including particle size, shape (relative (100) and (111) energies), and the number of simulated particles, were chosen based on an analysis of equimolar particles at 0.8 V versus RHE, the applied potential in simulations with fixed potential. The analysis is provided in Section S3.2 (Figs. S9–S19). In the presented results, the particles are 4 nm with equal (111) and (100) facet energies, where 10 equimolar particles yield a standard error below 0.01 on ${S}_{d}$ (Fig. S15) and approximately 3 at.% on the final (111) surface composition (Fig. S16), which is considered representative. The effect of increasing potential is shown in Fig. S20.

## Results and Discussion

### Stability Composition Space

**Fig. 3:**
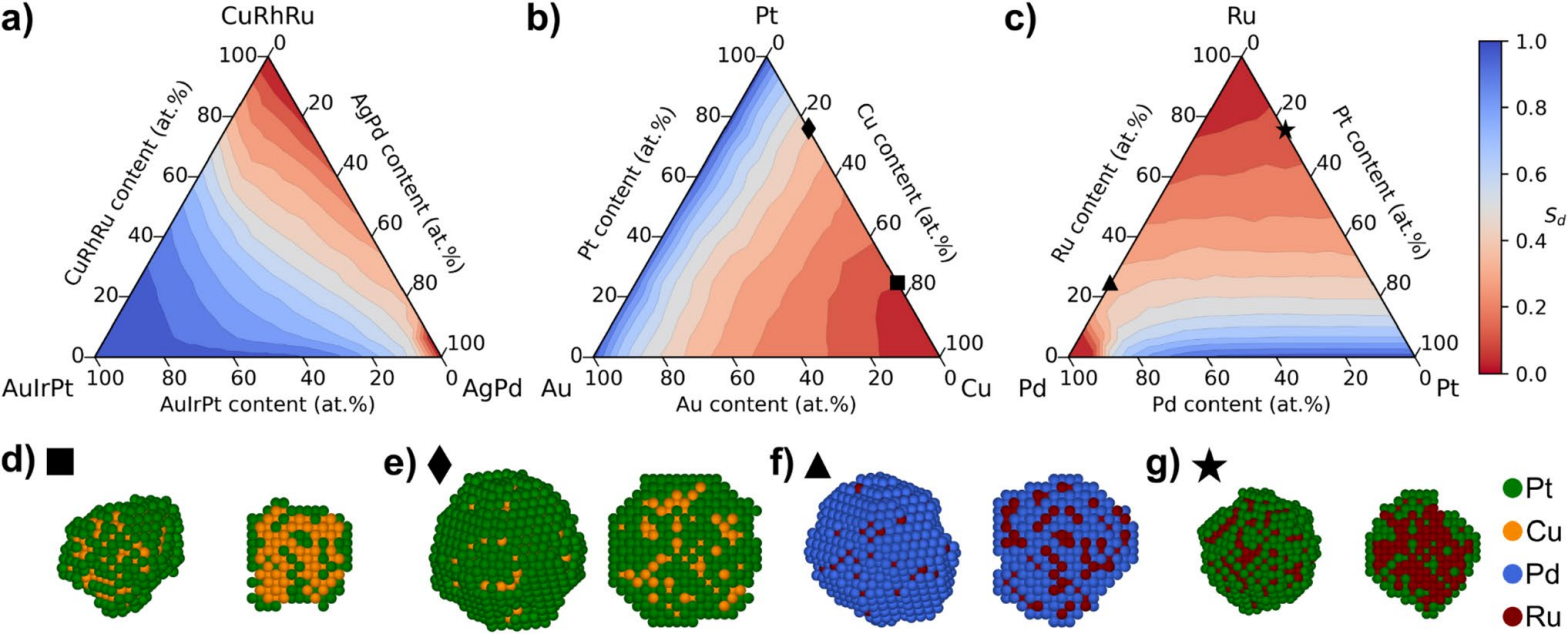
Composition spaces for ${S}_{d}$ as contour maps created from the simulated grid with particle examples. In **a** the 8-metal space is shown as a pseudo-ternary plot by combining molar fractions, showing the most stable composition on top where more are available. **b** and **c** show the ternary spaces of Au–Cu–Pt and Pd–Pt–Ru, respectively. **d**–**g** show the final particle (left) with a cross-section view (right) of one of the simulated particles for four selected compositions: Cu_75_Pt_25_, Cu_25_Pt_75_, Pd_75_Ru_25_, and Pt_25_Ru_75_, respectively

The composition space of Ag–Au–Cu–Ir–Pd–Pt–Rh–Ru was divided into a 1/16 molar fraction (6.25 at.%) incremental grid to investigate stability trends across compositions. Ten simulations of 4 nm particles at 0.8 V were performed for each grid composition, recording the average result. The resulting stability composition space is represented in Fig. 3 using contour maps. Figure 3a shows the entire 8-metal space in a pseudo-ternary composition space in which molar fractions are combined into three, grouped by the reduction potentials of the metals. Figure 3a unveils that in the presence of stable metals (${U}_{M}>U$), there are no local maxima due to a gradient towards the metals with higher reduction potential than the applied potential (Au, Ir, and Pt). Moreover, the regions of maximum stability on the grid (${S}_{d}=1$) span from the stable metals (see Table S5). Excluding the stable metals, i.e., considering the AgPd–CuRhRu edge, an internal maximum on the grid emerges (Pd_81.25_Rh_12.5_Ru_6.25_). The lack of maxima disconnected from stable metals trivializes the optimization of ${S}_{d}$ within this composition space. However, when combined with other objectives, such as catalytic activity and material cost, an array of Pareto optimal compositions may emerge. Thus, utilizing this stability model in multi-objective optimization will be of future interest.

To increase the interpretability of the stability composition space, two representative ternary subspaces, Au–Cu–Pt and Pd–Pt–Ru, are shown in Fig. 3b and c, respectively. Starting with the unstable Cu in Fig. 3b, adding the noble metals Pt and Au stabilizes the composition slowly. The similar stabilization rate between Pt and Au indicates that the nobility of the stabilizing metals may be less significant. Figure 3d and e, showing a resulting particle of Cu_75_Pt_25_ and Cu_25_Pt_75_, illustrate how alloying with noble metals stabilizes the particle relative to Cu. The dissolution process forms a Pt-layer leading to core–shell (PtCu@Pt) particles, aligning well with experimental observations [59, 60]. Moreover, it conforms in general with the widely studied Pt–M (M = Co, Fe, Cu, Ni) nanoparticles for ORR, where exposing Pt–M particles to corrosive environments is known to form Pt-skeleton particles, which are core–shell nanoparticles with a rough Pt surface layer depleted of the non-noble metal and a bulk core with retained composition [17, 22, 59, 61–63]. Within our model, the noble shell acts as a passivation layer, protecting the non-noble elements in the bulk from dissolution. Increasing the amount of Pt facilitates the formation of the Pt-layer resulting in less dissolved particles that retain more (111) atoms relative to Cu-Pt particles containing less Pt, which is deemed as more stable by the applied stability metric. Thus, the stability of a particle is determined by the formation of a stable layer.

In Fig. 3c, Pd is stabilized rapidly by adding Pt due to Pd’s reduction potential of 0.77 V being close to the applied potential of 0.8 V. Besides the stabilization from adding noble metals, results of interactive effects emerge on the Pt–Au and Pd–Ru edges. On the Pt-Au edge, where the constituting metals are deemed fully stable at the modelled conditions, the stability drops by up to 6% from mixing the elements (Fig. S21). Reversely on the Pd–Ru edge in Fig. 3c, mixing fully dissolvable elements increases stability. These observations are the results of interactive effects captured by the neighbor parameters in the regression model (Fig. 2b), which correlate with the relative surface energies of the elements, as shown in Fig. S5. The neighbor parameters reveal a zero-sum-like interaction where, in most cases, a stabilizing effect on atom A results in a destabilization of atom B for an A-B neighbor atoms pair. Thus, Au destabilizes Pt due to its lower surface energy, and thereby, the dissolution potential of a Pt atom surrounded by enough Au neighbors can decrease sufficiently to make the Pt atom soluble. In the case of the Pd–Ru edge, Ru effectively stabilizes Pd to elude surface exposure due to its high surface energy. Thus, adding a sufficient amount of Ru to Pd (approximately 25 at.%) results in particles that, while partly dissolved, persist at conditions where the individual constituent elements would otherwise dissolve. As a result, the dissolved particles form a core@shell nanoparticle structure of PdRu@Pd, as illustrated in Fig. 3f. The shell is, thus again, depleted by the readily dissolved element. The formed Pd shell with sub-surface Ru effectively acts as a passivation layer on a nanoparticle smaller than its initial state, which could be the basis of a potential post-synthesis design principle for creating ultra-small nanoparticles with increased stability for ORR, similar to dealloying for introducing porosity to enhance activity [52, 64]. However, the potential advantage may depend on the recoverability of dissolved precious metals. The principle of utilizing high surface energy metals, such as Ru, would also apply to Pt-alloys such as PtRu. Figure 3g shows a dissolved Pt_25_Ru_75_ particle displaying a core–shell structure, which aligns with recent findings for PtRu alloy particles [65]. Although Ru, like Cu, readily dissolves from the surface, Ru stabilizes the Pt atoms by increasing their dissolution potentials, resulting in a slightly higher stability.

The stability spaces reveal two distinct alloying strategies. The first involves stabilizing a composition using a noble element (${U}_{M}>U$) as a stabilizing agent. However, this strategy may inadvertently destabilize desired surface elements if the stabilizing agent has significantly lower surface energy. Secondly, an element with high relative surface energy can function as a stabilizing agent. Although this element may dissolve from the surface, its presence in the sub-surface layer enhances the dissolution potential of the surface atoms.

### Evolution in Composition Space Through Dissolution

**Fig. 4:**
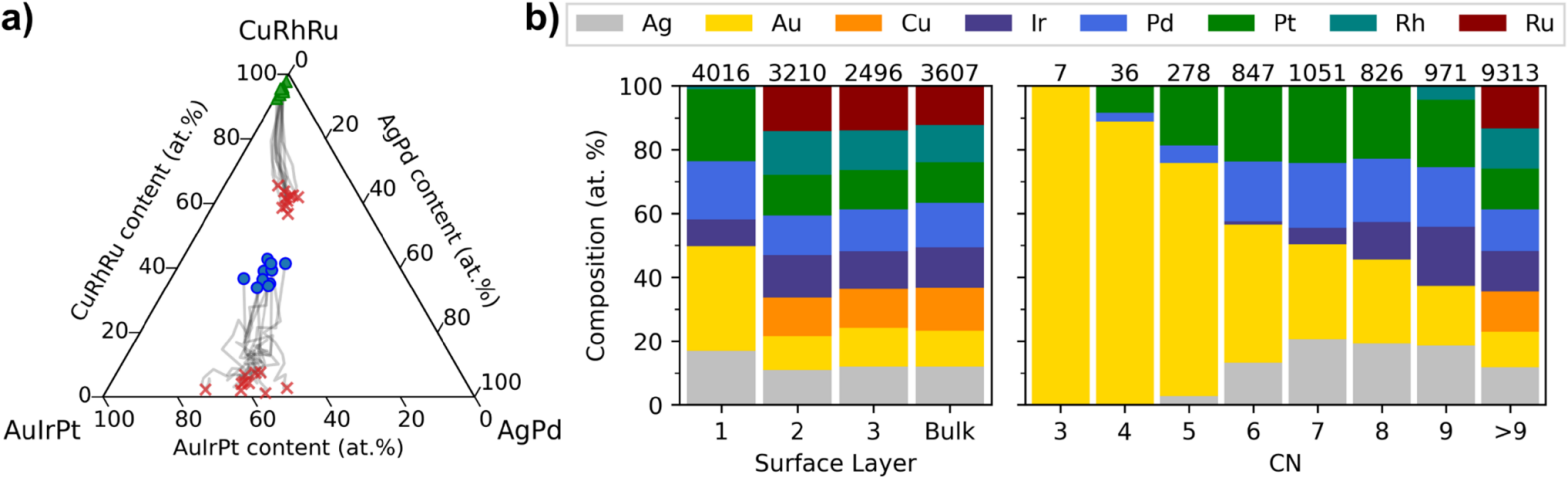
**a** Paths in composition space by each iteration of dissolution for ten equimolar octo-metallic particles. The paths originating in the blue circles and green triangles show the (111) surface and dissolved compositions, respectively, while the red crosses show the final composition. **b** Composition profiles of surface layers and coordination number (CN) showing the average compositions of the particles with the total number of atoms in each composition given above. “Bulk” refers to the atoms below the three layers (Color figure online)

The change in surface composition during operation is crucial for understanding and predicting the catalytic activity in designing new catalysts. In previous work, we uncovered paths of maximum activity as ridges in HEA composition space for ORR, from which we proposed designing catalysts that degrade along the ridges to retain activity [46]. Utilizing this strategy would require insights into how the surface changes during electrochemical degradation. We demonstrate the concept of following the evolution of the surface by recording the composition in each iteration of the simulation, thus tracing the path in composition space during dissolution. Figure 4a displays the initial- and final surface compositions connected by a path showing the surface composition after each iteration, i.e., batch, of dissolution for ten equimolar particles. Refer to Fig. S22 for the individual paths, Table S6 for the initial- and final compositions, and Fig. S23 for a visualization of the particles. Although the evolution of the (111) surface compositions is not direct, there is directionality towards a similar composition, with the discrepancies largely attributable to the lower statistics of only considering the (111) surface atoms. The change in the dissolved composition is more consistent, making it promising for experimental comparisons with ICP. Initially, the dissolution consists primarily of the non-noble metals Cu, Rh, and Ru, which leaves the stable atoms less coordinated and thereby vulnerable to dissolution, increasing the amount of the more stable elements in the dissolved composition with dissolution iterations. A study of the dissolution pathway in comparison with activity could be of future interest.

Figure 4b shows the final average composition by layer and CN of the particles in Fig. 4a. The layer profile demonstrates the preservation of the composition below the top surface layer, while the non-noble metals of Cu, Ru, and Rh predominantly dissolve from the surface. From CN 9 to 7, corresponding to the (111), (100), and (110) surfaces, the compositions remain similar, with only a gradual substitution of Ir for Au, likely due to differences in their surface energies. The comparable compositions for the three CNs indicate similar dissolution behavior and, thereby, similar trends in stability and activity during dissolution for the corresponding facets. Moreover, the profile shows that elements with low surface energy and high reduction potential, primarily Au, stabilize the particle by occupying undercoordinated sites, which is a well-documented stabilizing effect from Au in the literature that, furthermore, has been reported to be enhanced by the outward diffusion of Au, which occupies the vulnerable sites and shields dissolvable atoms [66–69]. Diffusion within the particle is unaccounted for in this study. Thus, within this model, Au is likely underrepresented in defect positions, possibly leading to underestimation of stability for Au-alloys. Reversely, Au in bulk will destabilize the surface atoms per the neighbor parameters. Therefore, stabilizing with Au, or likewise, could be more favorably achieved through galvanic replacement [70–72] of lower coordinated surface atoms, which additionally would be an efficient use of Au. We exemplify this approach for Pd–Au particles illustrated in Fig. S24. Replacing Pd with CN ≤ 6, 7, and 8 with Au increases the minimum dissolution potential from 0.77 V to 0.89, 0.94, and 1.14 V, respectively, making the particles fully stable below these potentials within the scope of this model. Comparing with ${S}_{d}<1$ at 0.8 V from stabilizing with increasing amounts of Au through alloying, as shown in Fig. S25, indicates the strategy of galvanic replacement is more effective.

**Fig. 5:**
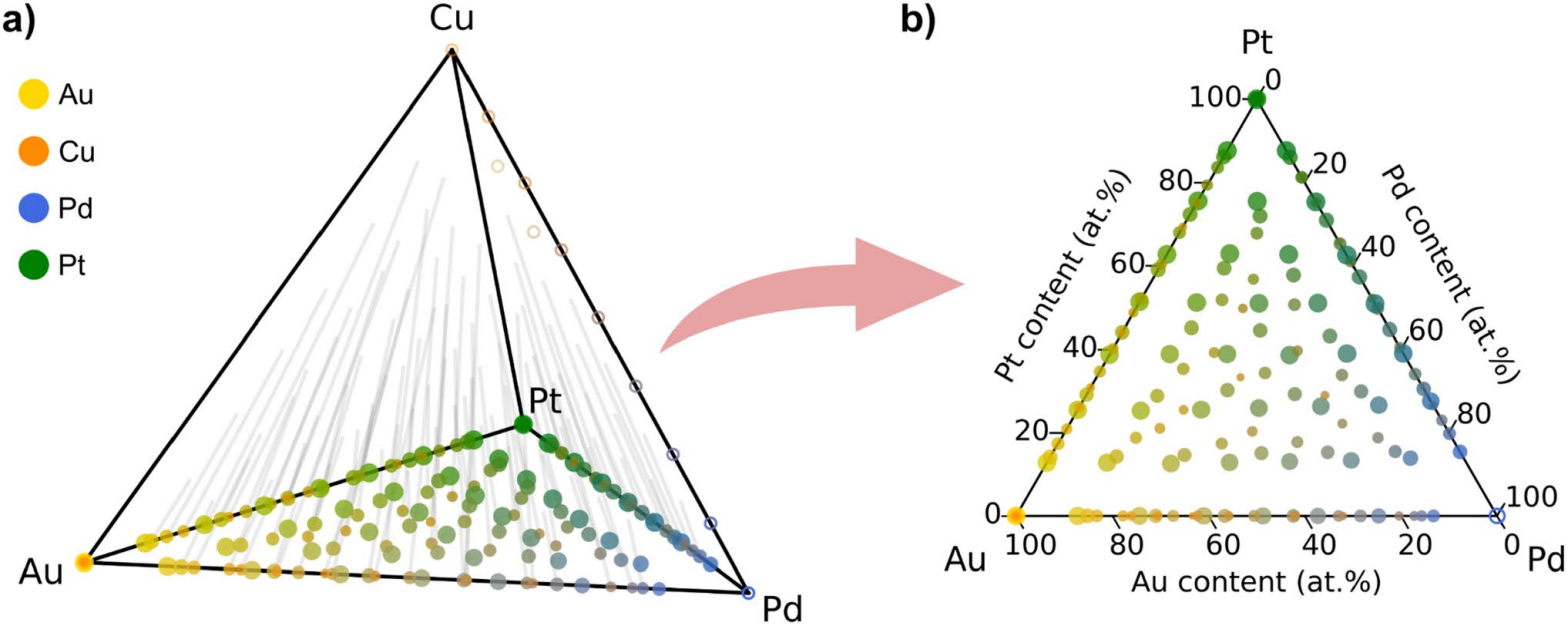
(111) Surface compositions after dissolution of Au–Cu–Pd–Pt alloy particles. **a** Shows the quaternary space, whereas **b** shows the Au–Pd–Pt subspace. The color denotes the bulk composition by a weighted average of the metal colors by composition, while the marker size scales with ${S}_{d}$. Unfilled markers represent compositions with ${S}_{d}=0$. The grey lines in **a** serve as a guide to the eye, connecting the initial- and final (111) surface compositions (Color figure online)

Figure 4a shows the evolution of the surface composition through composition space. Relating the final surface composition to its initial state reveals that a vast part of composition space may be unattainable for surface composition. Figure 5a illustrates this by showing the final (111) surface compositions of the grid simulations for the quaternary Au–Cu–Pd–Pt space (limited to 12.5 at.% grid compositions for intelligibility). As Cu completely dissolves from the surface, the surface composition devolves to the ternary Au–Pd–Pt subspace, as shown in Fig. 5b. The lines in Fig. 5a, connecting to the initial surface composition, follow the same directionality in composition space directly away from the Cu corner, approximately conserving the ratios between Au, Pd, and Pt.

### Model Limitations and Future Improvements

Although the presented dissolution model can capture experimental trends in predicting core–shell structures, the current framework provides no time dependency, which could challenge experimental comparisons where time is a factor, e.g., in electrochemical potential cycles.

In this demonstration, the model dissolves surface atoms in batches according to their predicted dissolution potential relative to the applied potential, which implicitly assumes an equal dissolution rate for all atoms. Although more computationally demanding, related inaccuracies and inconsistencies could be mitigated using the model implementation where only a single atom per iteration dissolves. Moreover, a constant metal-ion concentration is used as a proxy for the dissolution rate to compensate for solely thermodynamic energetics. Otherwise, concentrations that follow the dissolution would, upon initiation, yield an infinitely negative dissolution potential for all atoms. Thus, concentration effectively sets a reasonable limit on the rate of dissolution. However, the chosen concentration may affect the observed trends. If a metal’s reduction potential is relatively close to the applied potential, concentration may notably impact the result if the metal constitutes a large amount of the composition. Therefore, future work could involve including kinetic barriers for dissolution or determining appropriate concentrations through experiments. Additionally, dissolution mechanisms and re-deposition could be included through kinetics to capture their effect on the dissolution of alloy nanoparticles.

For a fast and straightforward particle relaxation scheme, the atoms were allowed to move to increase coordination, implying three assumptions: Increasing CN decreases energy independent of the atomic environment, each incremental increase in CN is equally favorable, and energetic barriers associated with moving to a neighboring position are negligible. Moreover, the model restricts the atoms to fcc lattice positions, dismissing any other surface or structure that could reduce the total energy. Only repositioning atoms to unoccupied neighboring lattice positions also disregards other means of particle relaxations driving surface segregation, such as increasing presence on the surface of elements with low surface energy. Thus, future endeavors in developing this model could entail a more detailed mechanism for particle relaxation. Doing so through DFT or molecular dynamics is infeasible for HEA compositional screening. However, machine-learning force fields and interatomic potentials, including atomic cluster expansion, could provide the necessary speedup to include more accurate particle relaxations and atomistic diffusion in future implementations [73–76]. It is also worth noting that the actual particle size and shape will depend on the synthesis, thus challenging a factual representation of the particle and its dependence on the composition within the simulations.

Oxide formation, adsorbates, and ions in the electrolyte are currently not accounted for but may impact dissolution [18, 25, 32, 77–79]. Their effect could, therefore, be of interest to include in future studies. Besides particle degradation driven by dissolution, mechanisms such as particle detachment, aggregation, and coalescence may play a considerable role in the loss of ECSA and catalytic activity [18, 26, 27, 80–82]. Such electrochemical degradation mechanisms, not captured by this model, may further challenge direct experimental comparisons with the model.

We have demonstrated this novel workflow for simulating dissolution using nanoparticles with acidic ORR as an example, as its corrosive electrochemical environment makes it an ideal case study. The methodology is applicable to other reactions and can be generalized to other nanomaterials given that the utilized metals are subject to dissolution within a relevant potential range. Appropriate examples include CO oxidation, which requires similar potentials as ORR [83], and the application of iron-group metals in alloys for hydrogen evolution or hydrogen oxidation reactions, which have also been studied for other catalyst HEA nanomaterials [84, 85]. Moreover, Cheng-Yu Wu et al. [84] demonstrated enhanced durability versus commercial Pt/C for an HEA containing Ru in potential cycles after forming a stable catalyst layer, which, according to our findings, could be due to sub-surface atoms, e.g., Ru stabilizing surface Pt atoms.

## Conclusion

In this study, we addressed the lack of fundamental and theoretical insights on the electrochemical stability of complex multi-metallic systems, such as high-entropy alloys, by developing a theoretical framework for simulating the dissolution of nanoparticles under electrochemical conditions. We demonstrated this methodology for the oxygen reduction reaction using the octo-metallic Ag–Au–Cu–Ir–Pd–Pt–Rh–Ru high-entropy alloy system.

The dissolution of a given particle was simulated by calculating the dissolution potentials of each surface atom depending on its local atomic environment, enabled by predicting the change in surface energy using a simple machine-learning regression model trained on density functional theory calculations. A stability parameter, measuring the number of (111) surface atoms before versus after the simulation, was presented to analyze stability trends in composition space through a grid search. Analyzing the stability composition space uncovered two stabilizing strategies through alloying: (1) Increasing the concentration of metals with reduction potential above the applied potential and (2) incorporating an element with a high relative surface energy. The dissolution model resulted in core–shell particles with shells depleted of elements with low dissolution potential in agreement with experimental studies on binary Pt-alloys. In effect, the dissolution proceeds until a stable layer forms, and its facilitation determines the size of the resulting particle. The noble metals stabilize the particle by constituting the protective shell and occupying the low coordinated positions. Meanwhile, metals with high surface energy situated in the sub-surface layer have a stabilizing effect by increasing the dissolution potential of the surface atoms.

Our findings show that the surface composition changes substantially during dissolution. We demonstrated that the dissolution model can follow the evolution of the surface while tracking the experimentally measurable dissolved composition, revealing traceable paths in composition space of both surface- and dissolved composition. Moreover, this analysis indicated that a large part of the composition space may be unretainable for surface compositions.

Besides supporting intuitive results, such as elements with low reduction potential depleting from the surface through dissolution, our demonstrations show that a model built on this framework gains insight into less intuitive outcomes, notably, how interactions between elements impact the degree to which a particle with a given composition dissolves and changes surface composition. Moreover, trends in the literature showing core–shell formation were reproducible despite the simplicity of the dissolution model used to demonstrate the presented framework. Further validation could entail experimentally verifying that elements with high surface energy in sub-surface layers can stabilize atoms on the surface. Utilizing the current model for other predictions than investigating trends is likely to have little validity. However, further development toward a more rigorous dissolution model within this framework could increase prediction accuracy. Combining a model for electrochemical stability, such as the one presented in this study, with current activity models and other relevant objectives in multi-objective optimization of high-entropy alloys is of great interest in establishing a fully fledged catalyst discovery platform.

## Supplementary Information

Below is the link to the electronic supplementary material.

Supplementary file1 (PDF 27925 KB)

## Data Availability

The archived repository containing data and scripts for reproduction can be downloaded from 10.17894/ucph.126293a2-481a-4368-b9e4-53a47794cf5a. The repository can also be accessed on GitHub (may be subject to change): https://github.com/catalyticmaterials/HEA-nanoparticle-dissolution-model
