# Supplementary material for: Electrochemical Dissolution: Paths in High-Entropy Alloy Composition Space
Source: High Entropy Alloy Mater. 2025 Apr 29;3(1):165–77. doi: 10.1007/s44210-025-00057-3 (PMC12257736; doi:10.1007/s44210-025-00057-3)
Supplement: Supplementary file 1 — Supplementary file1 (PDF 27925 KB) [file 44210_2025_57_MOESM1_ESM.pdf]

Supporting Information

Electrochemical Dissolution: Paths in High-Entropy Alloy Composition Space

Mads K. Plenge[1], Jack K. Pedersen[1], Luis A. Cipriano[1], and Jan Rossmeisl[1,*]

[1]Department of Chemistry, University of Copenhagen, Universitetsparken 5, 2100 København Ø, Denmark
[*]Corresponding author: jan.rossmeisl@chem.ku.dk

The archived repository containing data and scripts for reproduction can be downloaded from https://erda.ku.dk/archives/daa87fca2c14d96566aa932d74ac6193/published-archive.html.
The repository can also be accessed on GitHub (may be subject to change):
https://github.com/catalyticmaterials/HEA-nanoparticle-dissolution-model

Contents

S1 Density Functional Theory Calculations **S3**

S2 Energy Regression Model **S7**

S3 Dissolution Simulation **S10**
S3.1 Model Variations S10
S3.2 Dissolution Model Analysis on Equimolar Nanoparticles S10

S4 Stability Composition Space **S17**

S5 Evolution in Composition Space Through Dissolution **S18**

List of Figures

S1 DFT Example Structures S4
S2 CN Particle S5
S3 Single Metal DFT Results S6
S4 HEA DFT Results S7
S5 Parameter Correlation S8
S6 Dissolution Potential Parity Plot S8
S7 Dissolution Prediction Confusion Matrix S9
S8 Dissolution Model Variations S10
S9 Equimolar Surface Compositions S11
S10 Equimolar Dissolved Compositions S11
S11 Equimolar Stabilities S12
S12 Surface Composition Size Dependence S12
S13 Dissolved Composition Size Dependence S13
S14 Stability Size Dependence S13
S15 Stability error per sample number S14
S16 Surface Composition error per sample number S14
S17 Surface Composition Shape Dependence S15
S18 Dissolved Composition Shape Dependence S15
S19 Stability Shape Dependence S16
S20 Stability Across Potentials S16
S21 Au-Pt Stability S18
S22 Individual Evolutionary Composition Paths S19
S23 Equimolar Particles S20
S24 Au-Pd Galvanic Replacement S21
S25 Pd-Au Alloying S21

List of Tables

S1 Lattice Parameters and Bulk Energies S3
S2 Structures S3
S3 Kept and discarded calculations of ΔE for HEA. S6
S4 Model Parameters S7
S5 Maximum Regions S17
S6 Evolved Compositions S18

S1 Density Functional Theory Calculations

The density functional theory calculations were performed with GPAW [1] (version 23.9.1), and the atomic structures were constructed within The Atomic Simulation Environment (ASE) [2]. For all calculations the revised Perdew-Burke-Ernzerhof (RPBE) functional [3] was implemented. The constructed structures correspond to 3×3 supercells with 5 atomic layers with periodic boundary conditions in the lateral directions and a vacuum layer of 10 Å along the z-direction. The structures were constructed using the lattice parameters in Table S1. For HEA strucures the average lattice parameter was used. An overview of the different structures are given in Table S2 and Figure S1. The electron wave functions were treated with periodic plane-wave functions with an energy cut-off of 400 eV. k-points of (4,4,1) were uniformly sampled using Monkhorst-Pack sampling [4]. The structures were relaxed using BFGS-linesearch to a convergence criteria of a maximum force of 0.05 eV/Å.

Table S1: Calculated fcc lattice parameters and bulk energies (E_{bulk}). obtained using the 'StrainFilter' in the ASE module, where the unit cell is relaxed using a stress tensor times volume as force [2]. The filter is imposed on an fcc bulk atom with periodic boundary conditions and relaxed using the integrated BFGS algorithm with a convergence criteria of 0.003 eV/Å. The lattice parameters were calculated with an E_{cut} of 1000 eV. The energies were calculated at the chosen E_{cut} level of 400 eV.

Metal	Lattice Parameter [Å]	E_{bulk} [eV]
Ag	4.2124	-2.316
Au	4.2142	-2.558
Cu	3.6898	-3.041
Ir	3.8854	-8.727
Pd	3.9841	-3.219
Pt	3.9957	-5.759
Rh	3.8676	-6.638
Ru	3.8303	-8.615

Table S2: Descriptions for the introduced defects for each coordination number (CN).

CN	Facet	Surface	Defect
3	111	terrace	adatom
4	100	terrace	adatom
5	211	edge	adatom
6	211	kink	vacancy
7	211	edge	vacancy
8	100	terrace	vacancy
9	111	terrace	vacancy

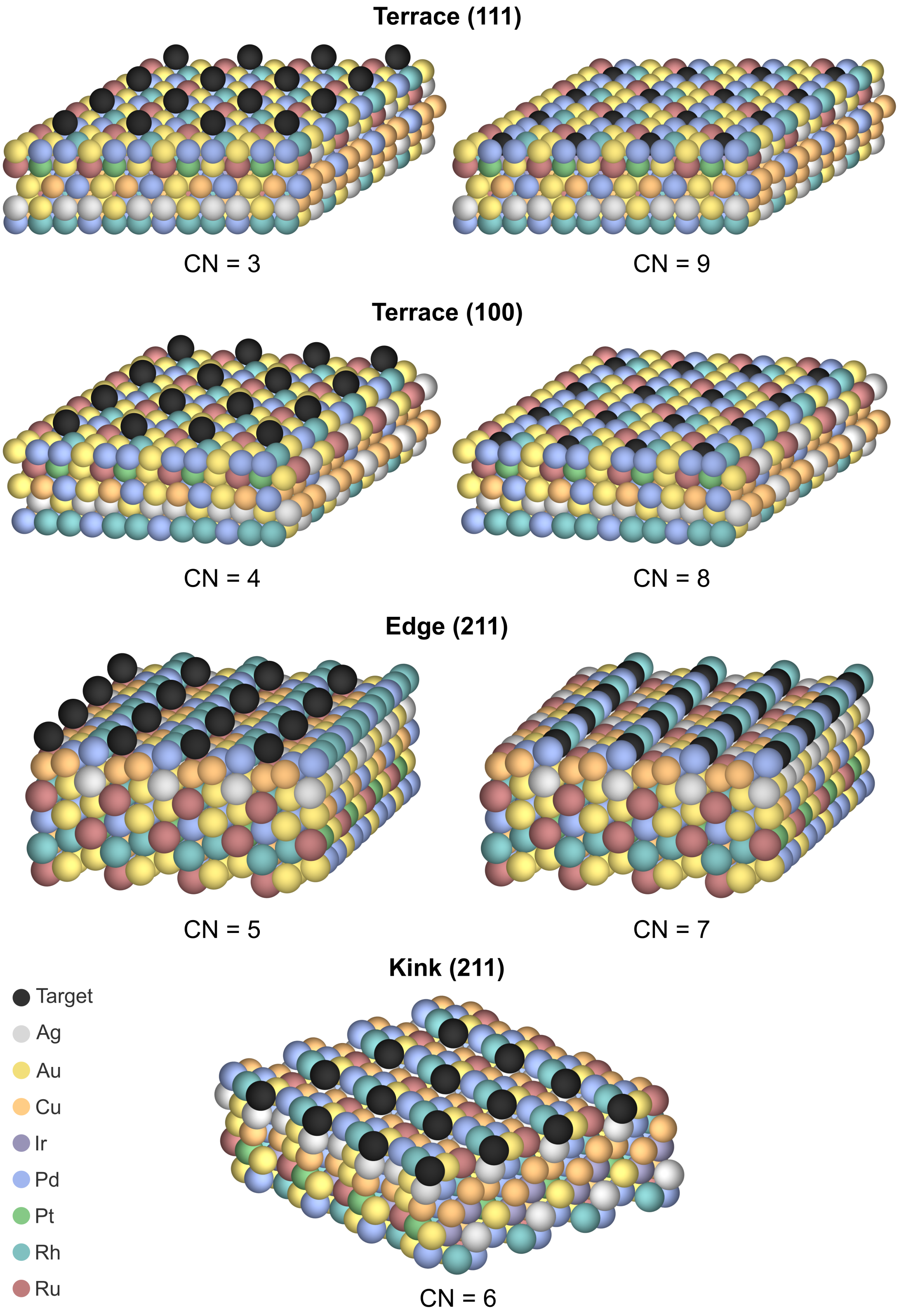

Figure S1: Example of a calculated HEA structure for each CN with the target atom highlighted in black.

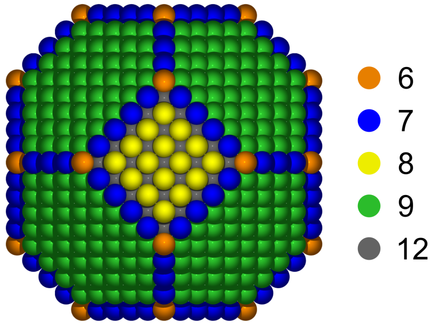

Figure S2: Coordination numbers of initial 4 nm particle.

Change in energy ΔE for the single metal structures are displayed in Figure S3. For HEA structures, in some cases, the structure after relaxation may deviate significantly from the modelled structure, especially the ones carrying defects. Observed deviations include: (111) adatoms moving to hcp sites, subsurface Au moving to fill surface vacancy leaving a bulk vacancy, (100) ad atoms (primarily Ir) moving into the surface layer disrupting the (100) structure, and cases of rearrangement of multiple or all surface atoms. As these cases not only deviated from the modelled surface, but also between the dissolved and non-dissolved slab making them incomparable, they are treated as outliers. The surface deviations were filtered from the data by not allowing any atom to have move more than $\frac{1}{2}$ of the interatomic distance upon initialization of the relaxation. This filter did not include adatoms. The data was also filtered by requiring the CN of the target atom to be in accordance with the modelled surface (see Table S2). The adatoms were thus given a larger tolerance in deviating from their original position, but had to retain their CN. However, for (111) adatoms, their final position was ensured to remain in an fcc position by requiring that the closest three atoms constituted an fcc position, which was used in place of the CN requirement. CN and neighbors were defined using ASE neighborlist with cutoff radius of $\frac{1}{2}$ an inter-atomic distance defined from the size of the unit cell. ΔE for HEA data after data treatment is shown in Figure S4. Kept and discarded calculations are listed in Table S3.

The single metal results, displayed in Figure S3, exhibit a linear trend of increase in ΔE with increasing CN. The removal of a kink atom on a metal surface has $\Delta E = 0$ in agreement with Greeley[5], i.e. no change in energy as the surface structure doesn't change, because removing a kink atom (CN=6), leaves a new kink atom. Consequently, for kink atoms in single metal particles, the dissolution potential depends solely on the reduction potential. Therefore, if the applied potential is above the metal's reduction potential, the kink atoms will dissolve in each iteration of dissolution in the model, resulting in a complete dissolution of the single metal particle at the reduction potential. However, this straightforward scenario applies only to particles consisting of a single metal. From the variety in configurations for HEA structures, distributions of ΔE emerges (Figure S4).

Table S3: Kept and discarded calculations of ΔE for HEA.

Surface	CN	Kept	Discarded
(111)	3	541 (68%)	259 (32%)
	9	782 (98%)	18 (2%)
(100)	4	668 (84%)	132 (16%)
	8	722 (90%)	78 (10%)
Edge	5	727 (91%)	73 (9%)
	7	758 (95%)	42 (5%)
Kink	6	741 (93%)	59 (7%)

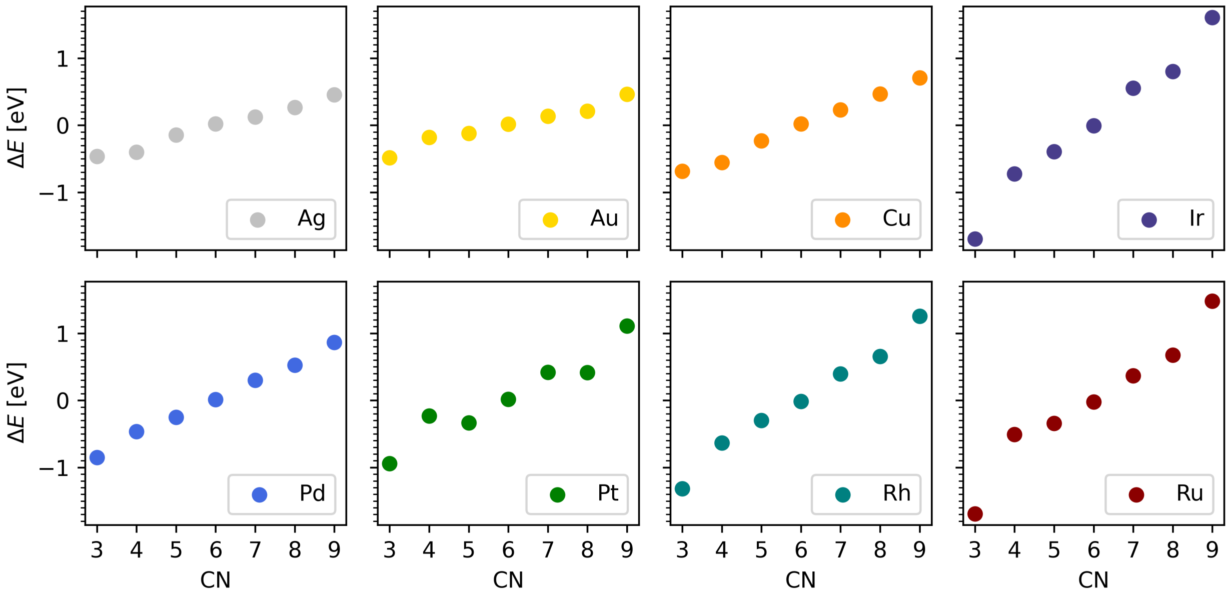

Figure S3: ΔE for single metal structures

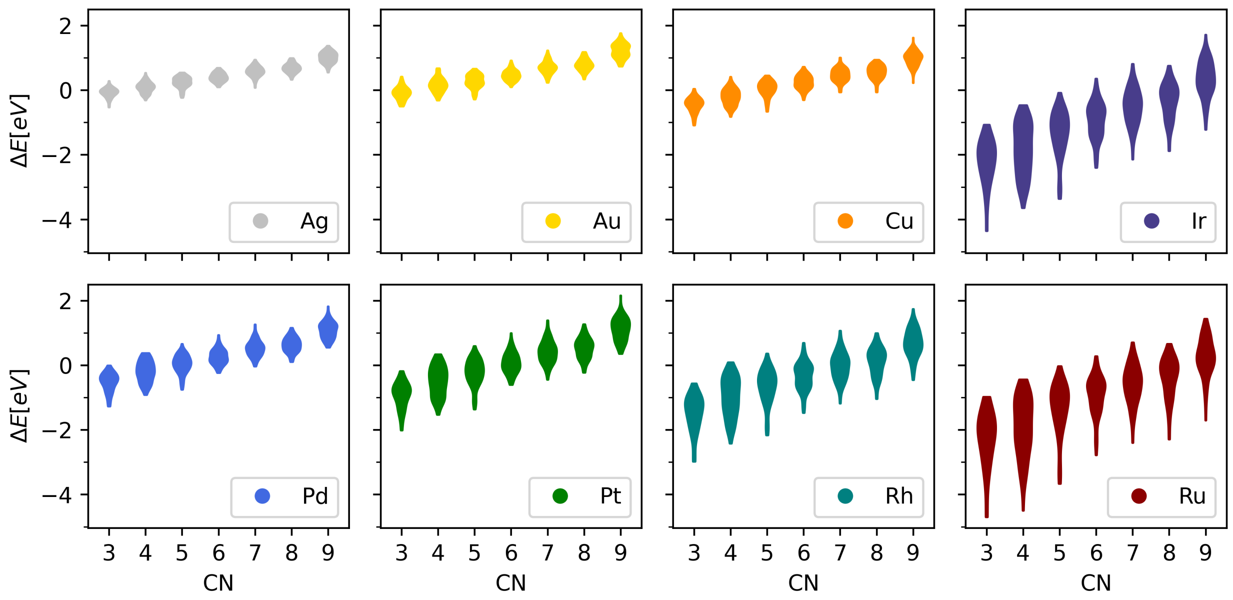

Figure S4: ΔE for HEA structures

S2 Energy Regression Model

Table S4: Model parameters (in eV).

	Target	Ag	Au	Cu	Ir	Pd	Pt	Rh	Ru
CN Parameters	3	-0.48	-0.57	-0.71	-1.34	-0.78	-1.02	-1.10	-1.28
	4	-0.27	-0.26	-0.37	-0.56	-0.38	-0.45	-0.51	-0.54
	5	-0.15	-0.21	-0.21	-0.34	-0.25	-0.33	-0.35	-0.31
	6	0.00	0.00	0.00	0.00	0.00	0.00	0.00	0.00
	7	0.18	0.23	0.22	0.54	0.27	0.35	0.33	0.49
	8	0.29	0.32	0.32	0.74	0.40	0.48	0.51	0.71
	9	0.60	0.74	0.72	1.42	0.83	1.06	1.06	1.36
Neighbor Parameters	Ag	0.00	0.14	-0.33	-2.52	-0.32	-0.69	-1.52	-2.85
	Au	0.12	0.00	-0.13	-2.29	-0.28	-0.77	-1.30	-2.31
	Cu	0.25	0.45	0.00	-1.52	0.12	-0.07	-0.79	-1.86
	Ir	0.65	0.70	0.70	0.00	0.84	0.84	0.75	0.50
	Pd	0.46	0.47	0.22	-1.51	0.00	-0.24	-0.81	-1.54
	Pt	0.57	0.45	0.48	-1.00	0.28	0.00	-0.35	-0.84
	Rh	0.54	0.66	0.42	-0.45	0.43	0.35	0.00	-0.42
	Ru	0.54	0.82	0.49	0.45	0.81	0.92	0.75	0.00

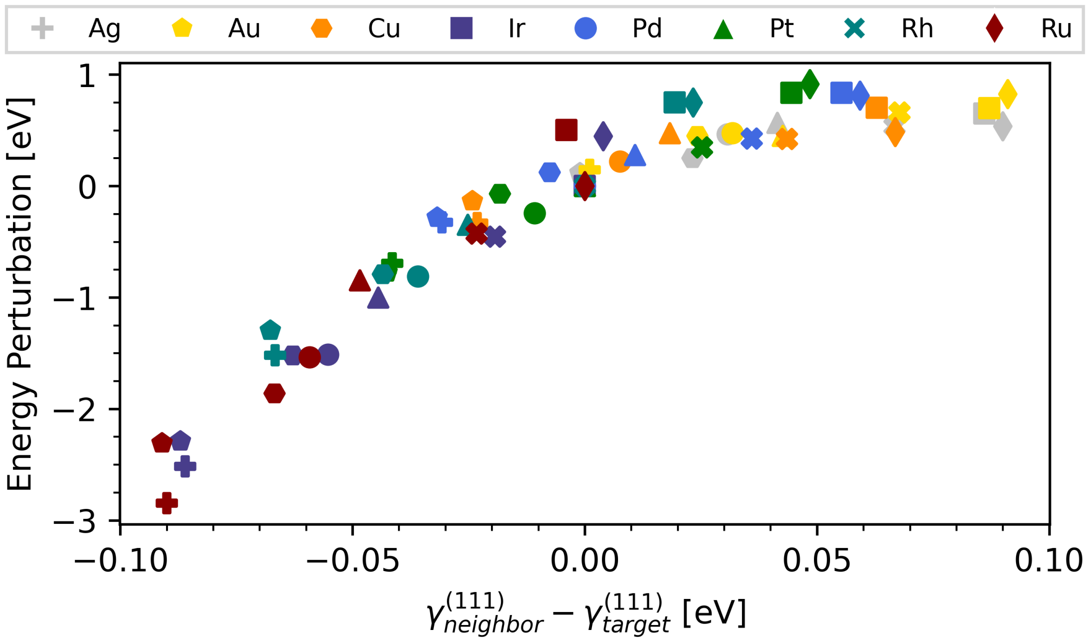

Figure S5: Relationship between neighbor parameters and relative (111) surface energies ($\gamma^{(111)}$) given by $\gamma^{(111)} = \frac{E_{slab}^{(111)} - E_{bulk} \cdot N_{atoms}}{2A}$, where $E_{slab}^{(111)}$ is the energy of the (111) metal slab, E_{bulk} is the bulk energy of the metal, N_{atoms} is the number of atoms in the slab, and A is the surface area[6]. Marker color represents the target metal and the marker symbol represents the neighbor metal.

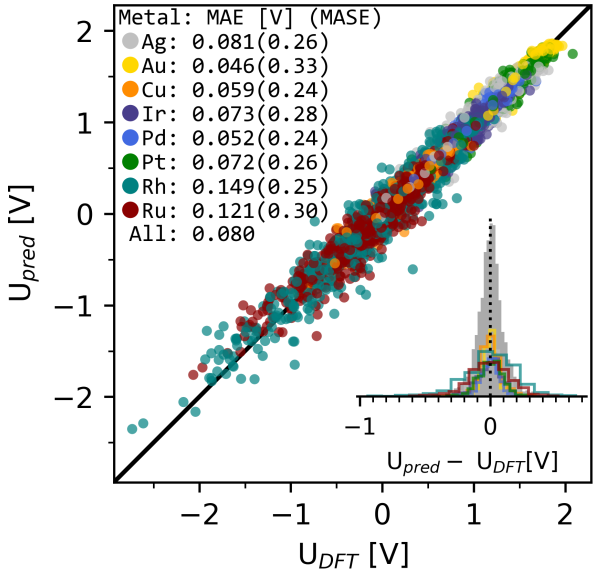

Figure S6: Parity plot of dissolution potentials from energies predicted with leave-one-out cross-validation. The inset shows the error distribution of all (filled grey) and by target metal (colored lines). The dissolution potentials are calculated assuming a metal concentration of 10^{-6} M.

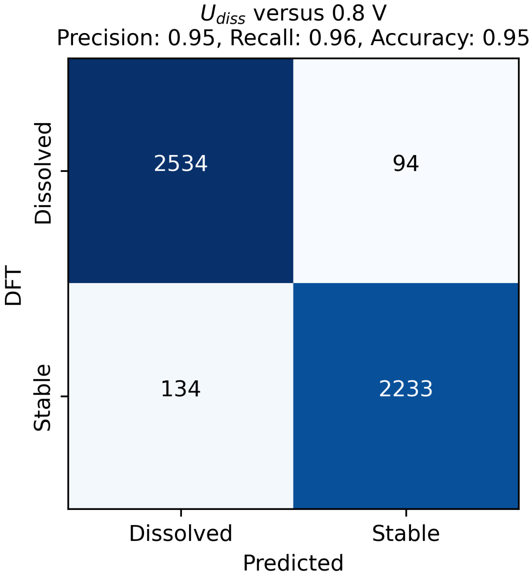

Figure S7: Confusion matrix comparing predicted versus DFT calculated dissolution potentials versus 0.8 V. Dissolved (positive) denotes dissolution potentials below 0.8 V.

S3 Dissolution Simulation

S3.1 Model Variations

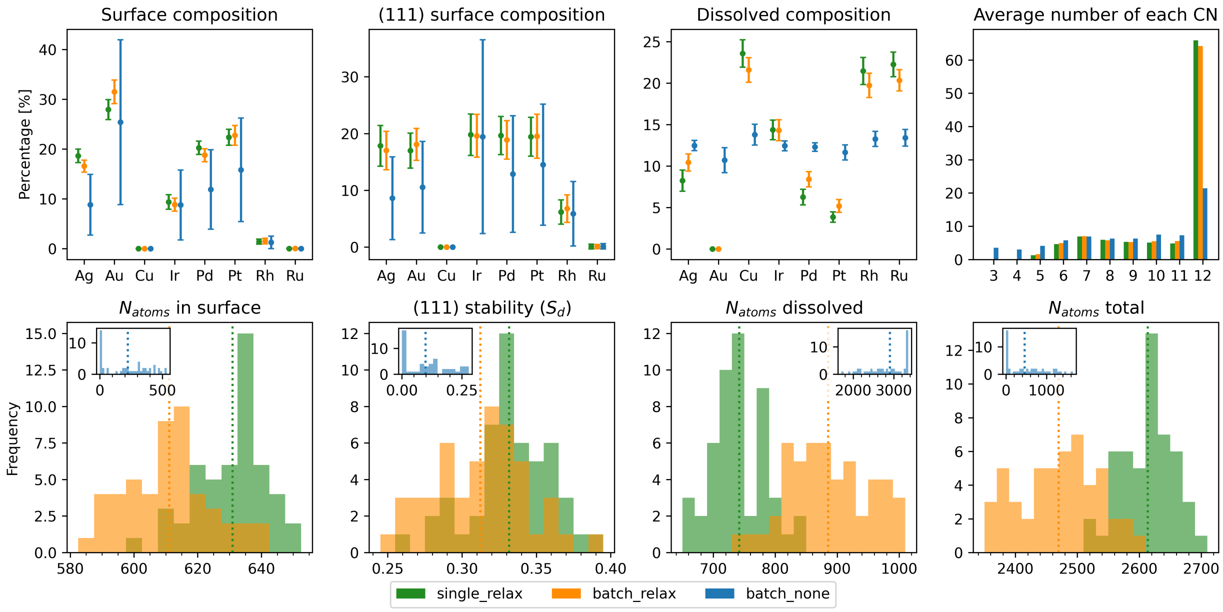

Figure S8: Results from of simulations on 50 different 5 nm equimolar particles using three variations of the model. 'single' versus 'batch refers to a single versus several atoms influenced between updating CN and U_{diss} of the atoms. batch_none: batch dissolution with no relaxation scheme. All atoms with $U_{diss} < U$ is dissolved in the current iteration, whereafter U_{diss} of all atoms are are updated. batch_relax: Batch dissolution with batch CN relaxation scheme between dissolutions where atoms are moved in batches to increase CN. The CNs are updated after each moved batch. Single_relax: The atom with the lowest dissolution potential is dissolved in each iteration of dissolution. In between dissolution, one atoms at a time, the one with highest improvement in CN, can change position.

S3.2 Dissolution Model Analysis on Equimolar Nanoparticles

The model, with batch dissolution and CN batch relaxation, is shown to provide normally distributed results on compositions and S_d (figures S9, S10, and S11). The mean (111) surface composition is consistent across particle sizes, but the dissolution composition varies slightly and S_d increases with particle size (figures S12, S13,S14, and S15). For all three quantities, the variance decreases with increasing particle size. A representable particle size or size distribution should be used in experimental comparisons. The (111) surface- and dissolved compositions are independent of particle shape from varying facet surface energies although affecting S_d (figures S17, S18, and S19). Therefore, equal facet energy will be used in constructing the particles.

Constant size, shape, and potential

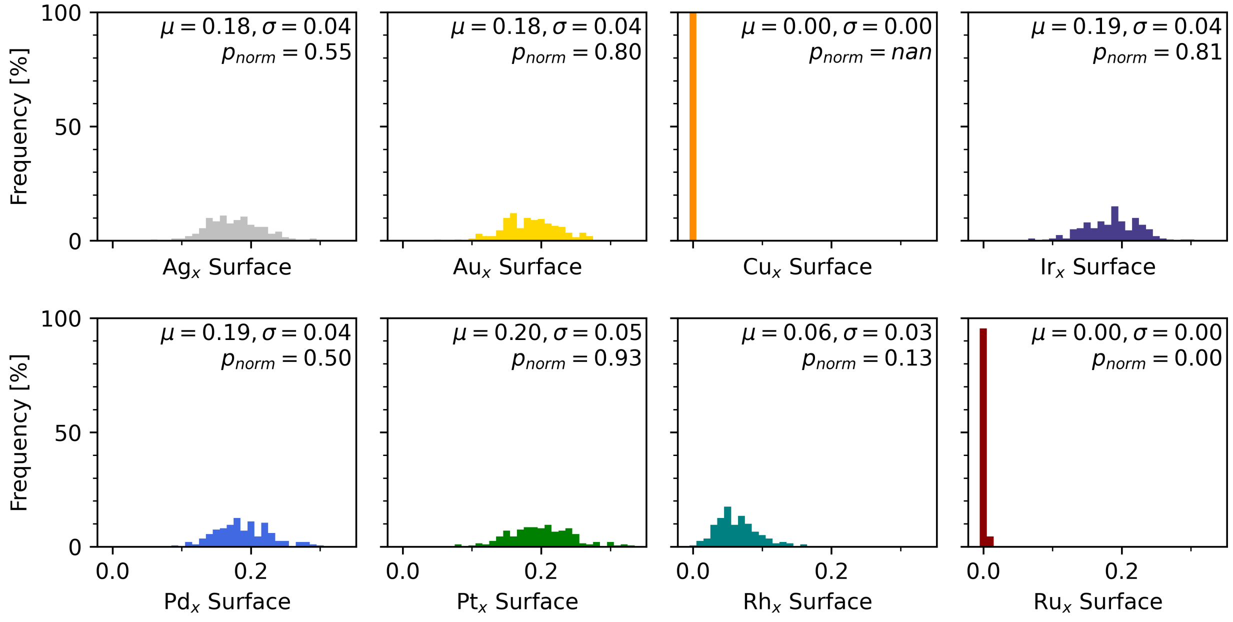

Figure S9: Distribution of (111) surface composition of 200 simulations of 4 nm equimolar AgAuCuIrPdPtRhRu particles at U=0.8 V.

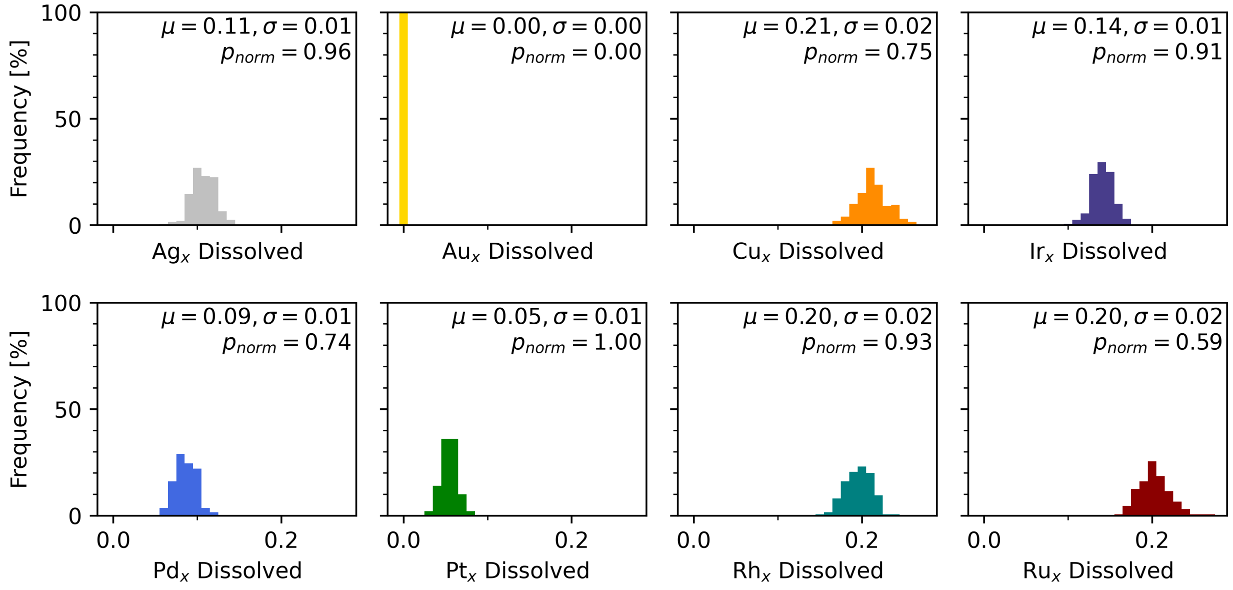

Figure S10: Distribution of dissolved composition of 200 simulations of 4nm nm equimolar AgAuCuIrPdPtRhRu particles at U=0.8 V.

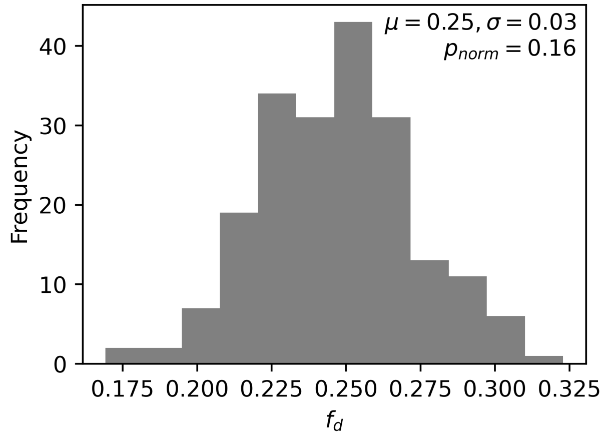

Figure S11: Distribution of stability parameter (S_d) from 200 simulations of 4 nm equimolar AgAuCuIrPdPtRhRu particles at U=0.8 V.

Size Dependence

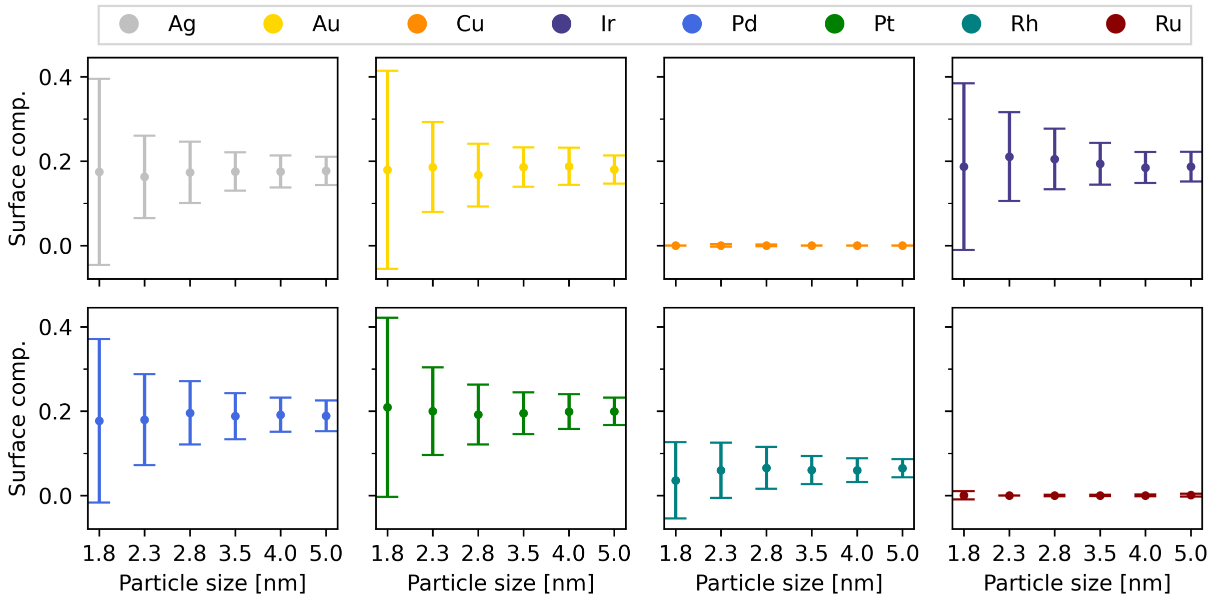

Figure S12: Size dependence of (111) surface composition.

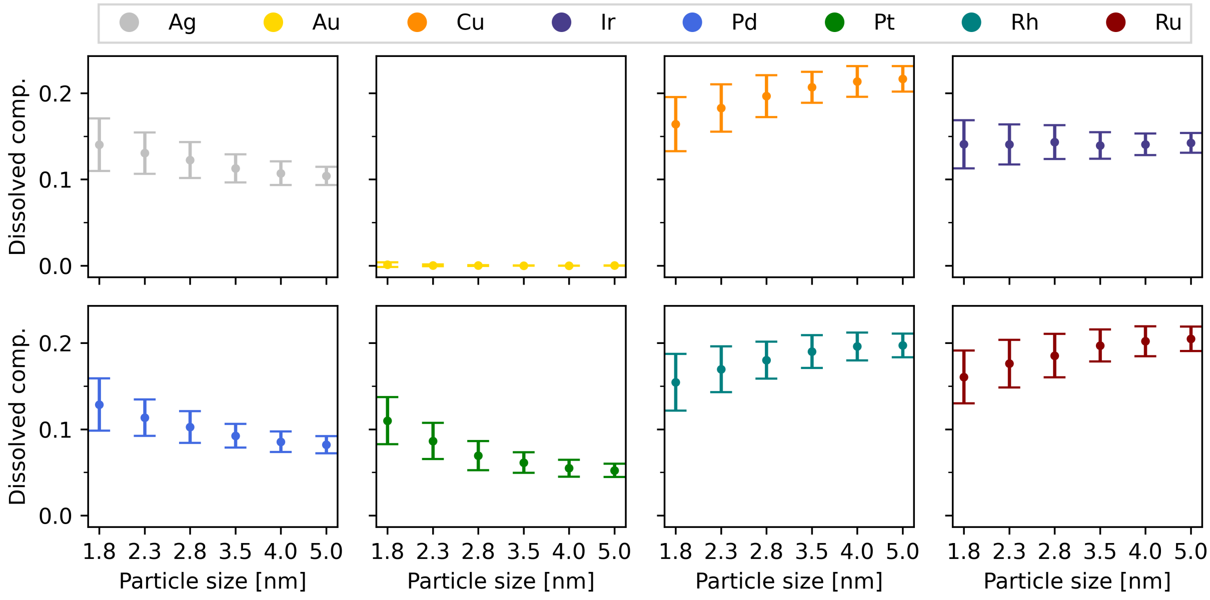

Figure S13: Size dependence of dissolved composition.

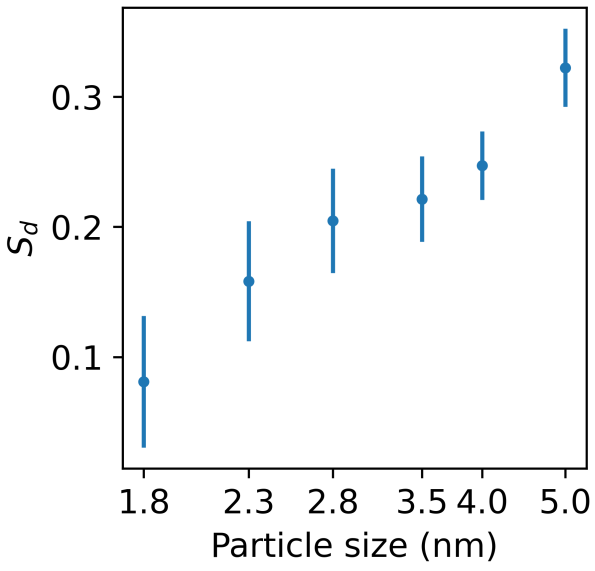

Figure S14: Size dependence of stability parameter (S_d).

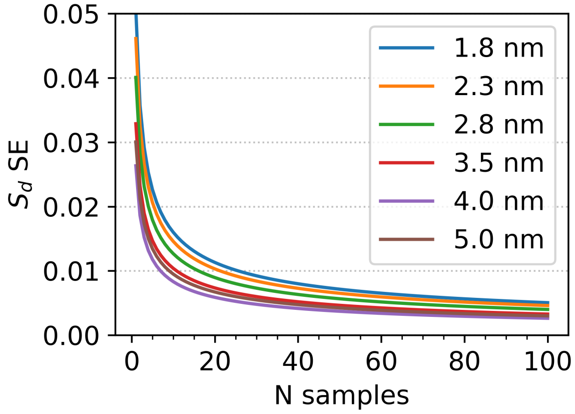

Figure S15: Standard error on estimated stability (S_d) as function of number of particles. The standard deviation of composition is estimated by the square root of the sum of variances for each metal component.

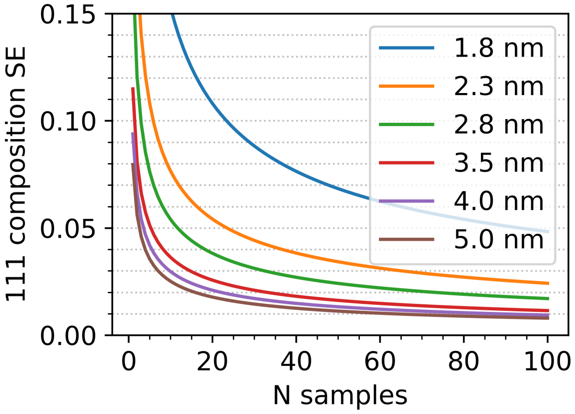

Figure S16: Standard error on estimated (111) composition after dissolution as function of number of particles.

Shape Dependence

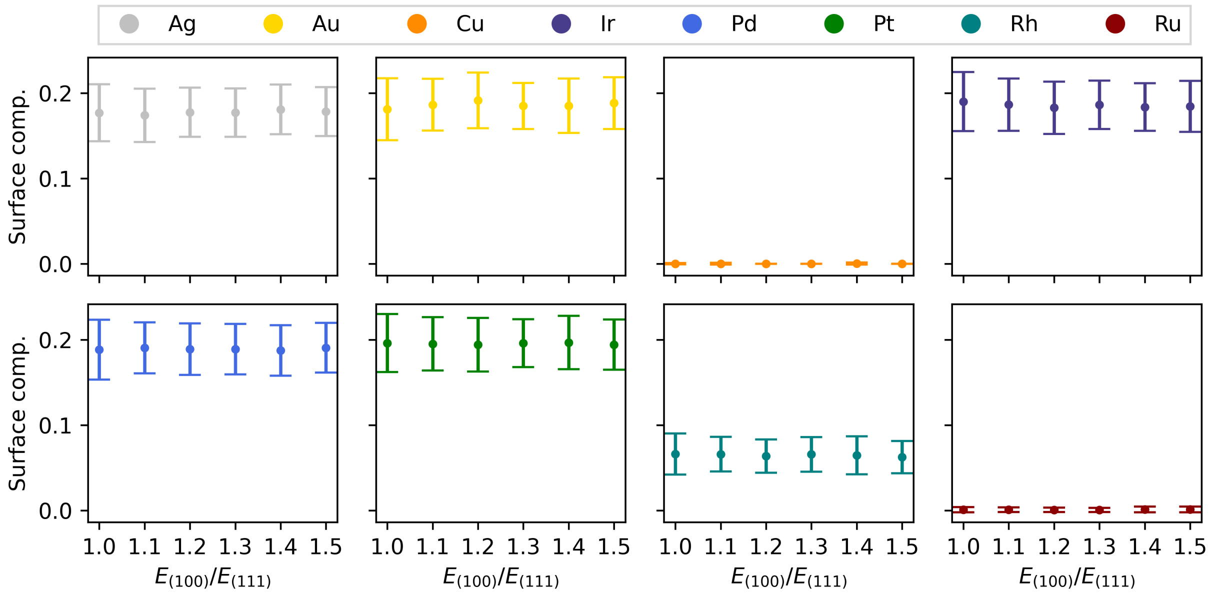

Figure S17: Shape dependence of (111) surface composition.

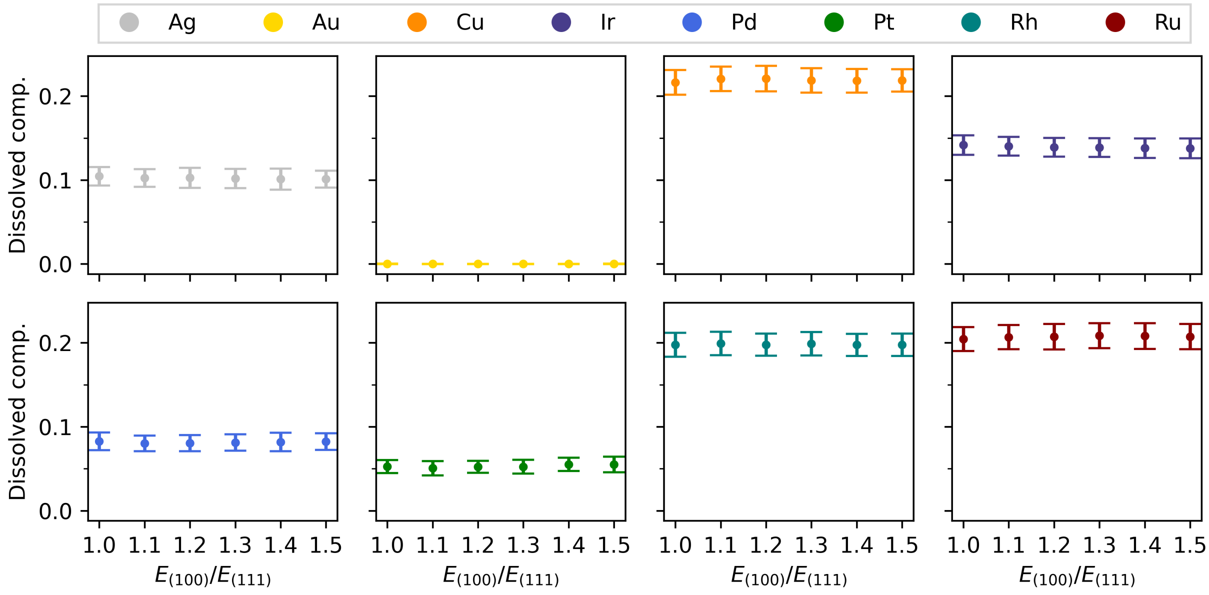

Figure S18: Shape dependence of dissolved composition.

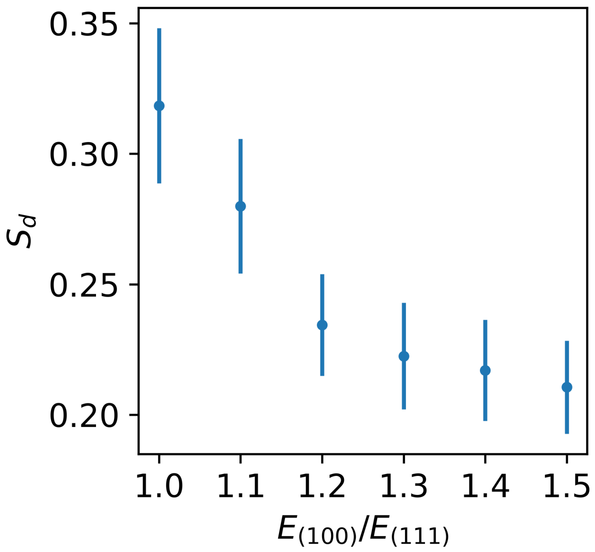

Figure S19: Shape dependence of stability parameter (S_d).

Potential Dependence

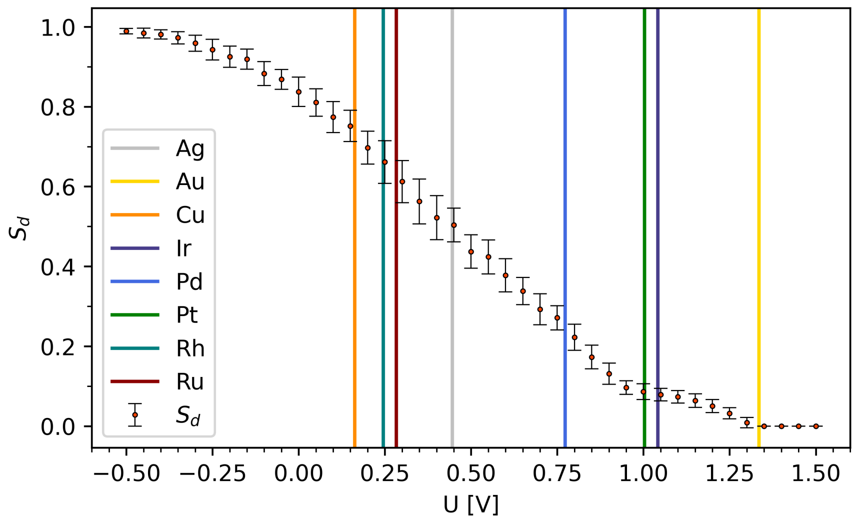

Figure S20: Stability (S_d) of 4 nm equimolar particles at increasing potentials. Lines mark the metals' dissolution potential.

S4 Stability Composition Space

Table S5: Regions of maxima on the composition grid. A grid maximum is characterized by having equal or above stability of all neighboring grid points. The regions are defined as being connected by a grid interval, i.e. neighboring grid compositions. The regions are denoted by a number and, if applicable, its included single metal. Compositions are given in at. %.

Region	Sd	Ag	Au	Cu	Ir	Pd	Pt	Rh	Ru
1 (Au)	1.00	0.0	100.0	0.0	0.0	0.0	0.0	0.0	0.0
2 (Ir)	1.00	0.0	0.0	0.0	100.0	0.0	0.0	0.0	0.0
		0.0	0.0	0.0	93.8	6.2	0.0	0.0	0.0
		0.0	0.0	0.0	93.8	0.0	6.2	0.0	0.0
		0.0	0.0	0.0	87.5	6.2	6.2	0.0	0.0
		0.0	0.0	0.0	87.5	0.0	12.5	0.0	0.0
		0.0	0.0	0.0	81.2	0.0	18.8	0.0	0.0
		0.0	0.0	0.0	75.0	0.0	25.0	0.0	0.0
3 (Pt)	1.00	0.0	0.0	0.0	0.0	0.0	100.0	0.0	0.0
		6.2	0.0	0.0	0.0	0.0	93.8	0.0	0.0
		0.0	6.2	0.0	0.0	0.0	93.8	0.0	0.0
		0.0	0.0	0.0	0.0	6.2	93.8	0.0	0.0
		12.5	0.0	0.0	0.0	0.0	87.5	0.0	0.0
		6.2	6.2	0.0	0.0	0.0	87.5	0.0	0.0
		6.2	0.0	0.0	0.0	6.2	87.5	0.0	0.0
		0.0	12.5	0.0	0.0	0.0	87.5	0.0	0.0
		0.0	6.2	0.0	0.0	6.2	87.5	0.0	0.0
		0.0	0.0	0.0	0.0	12.5	87.5	0.0	0.0
		6.2	0.0	0.0	0.0	12.5	81.2	0.0	0.0
		0.0	6.2	0.0	0.0	12.5	81.2	0.0	0.0
		0.0	0.0	0.0	0.0	18.8	81.2	0.0	0.0
		0.0	0.0	0.0	0.0	25.0	75.0	0.0	0.0
		0.0	0.0	0.0	0.0	31.2	68.8	0.0	0.0
4*	0.94	0.0	75.0	0.0	0.0	0.0	25.0	0.0	0.0

* The one disconnected local grid maximum $Au_{25}Pt_{75}$, visible on Figure S21, is not regarded to be significant as its improvement in S_d is 0.001 relative to surrounding compositions, which is well below the estimated uncertainty of 0.01, and moreover, it is positioned in a local minimum.

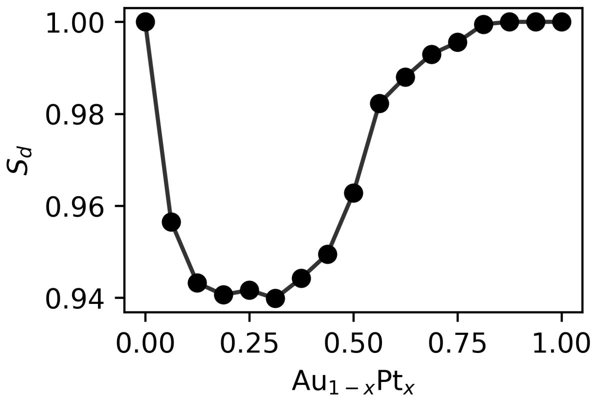

Figure S21: Stability along Au-Pt edge.

S5 Evolution in Composition Space Through Dissolution

Table S6: Compositions (in at. %) of samples in Figure 4 showing the initial and final (111) surface composition as well as the final dissolved composition.

	Sample	1	2	3	4	5	6	7	8	9	10
Initial (111)	Ag	13.0	12.8	12.5	11.7	13.3	12.2	13.3	12.0	14.8	7.5
	Au	14.1	11.7	12.5	12.2	12.0	13.0	10.2	12.8	12.0	12.8
	Cu	14.3	11.7	12.8	14.1	11.7	14.1	13.3	15.6	12.0	10.9
	Ir	10.7	10.7	13.0	12.5	14.6	11.5	7.8	11.5	12.0	14.8
	Pd	10.7	13.0	14.8	13.0	11.2	10.7	15.1	12.5	12.5	12.0
	Pt	12.5	12.5	12.0	14.1	15.1	9.9	12.2	9.9	14.3	16.2
	Rh	10.4	13.0	12.0	10.9	12.2	14.8	14.1	12.0	11.2	13.8
	Ru	14.3	14.6	10.4	11.5	9.9	13.8	14.1	13.8	11.2	12.0
Final (111)	Ag	21.3	16.4	20.2	18.6	21.2	19.8	19.5	23.2	17.0	9.2
	Au	18.0	19.8	18.1	18.6	12.1	17.0	24.1	13.0	20.4	26.4
	Cu	0.0	0.0	0.0	0.0	0.0	0.0	0.0	0.0	0.0	0.0
	Ir	16.9	19.8	20.2	15.5	20.2	13.2	19.5	18.5	21.6	19.5
	Pd	13.5	20.7	17.0	17.5	14.1	18.9	16.1	25.0	26.1	17.2
	Pt	23.6	19.0	17.0	27.8	28.3	23.6	16.1	17.6	13.6	25.3
	Rh	6.7	4.3	7.4	2.1	4.0	7.5	4.6	2.8	1.1	2.3
	Ru	0.0	0.0	0.0	0.0	0.0	0.0	0.0	0.0	0.0	0.0
Dissolved	Ag	10.9	9.6	13.4	6.4	11.3	10.0	10.8	8.9	11.2	10.0
	Au	0.0	0.0	0.0	0.0	0.0	0.0	0.0	0.0	0.0	0.0
	Cu	22.5	19.2	22.0	23.8	19.9	20.7	22.6	22.5	21.9	21.0
	Ir	14.4	12.4	15.1	15.1	14.8	14.0	10.3	15.0	14.0	18.0
	Pd	7.4	9.6	8.0	7.9	8.5	9.1	10.7	7.9	8.6	9.0
	Pt	5.6	6.0	6.8	5.0	6.2	6.0	6.2	4.4	3.7	4.4
	Rh	17.7	21.0	14.9	19.0	19.0	20.9	18.0	20.4	20.6	17.6
	Ru	21.4	22.2	19.8	22.8	20.3	19.3	21.3	20.8	20.0	20.0
	S_d	23.2	30.2	24.5	25.3	25.8	27.6	22.7	28.1	22.9	22.7

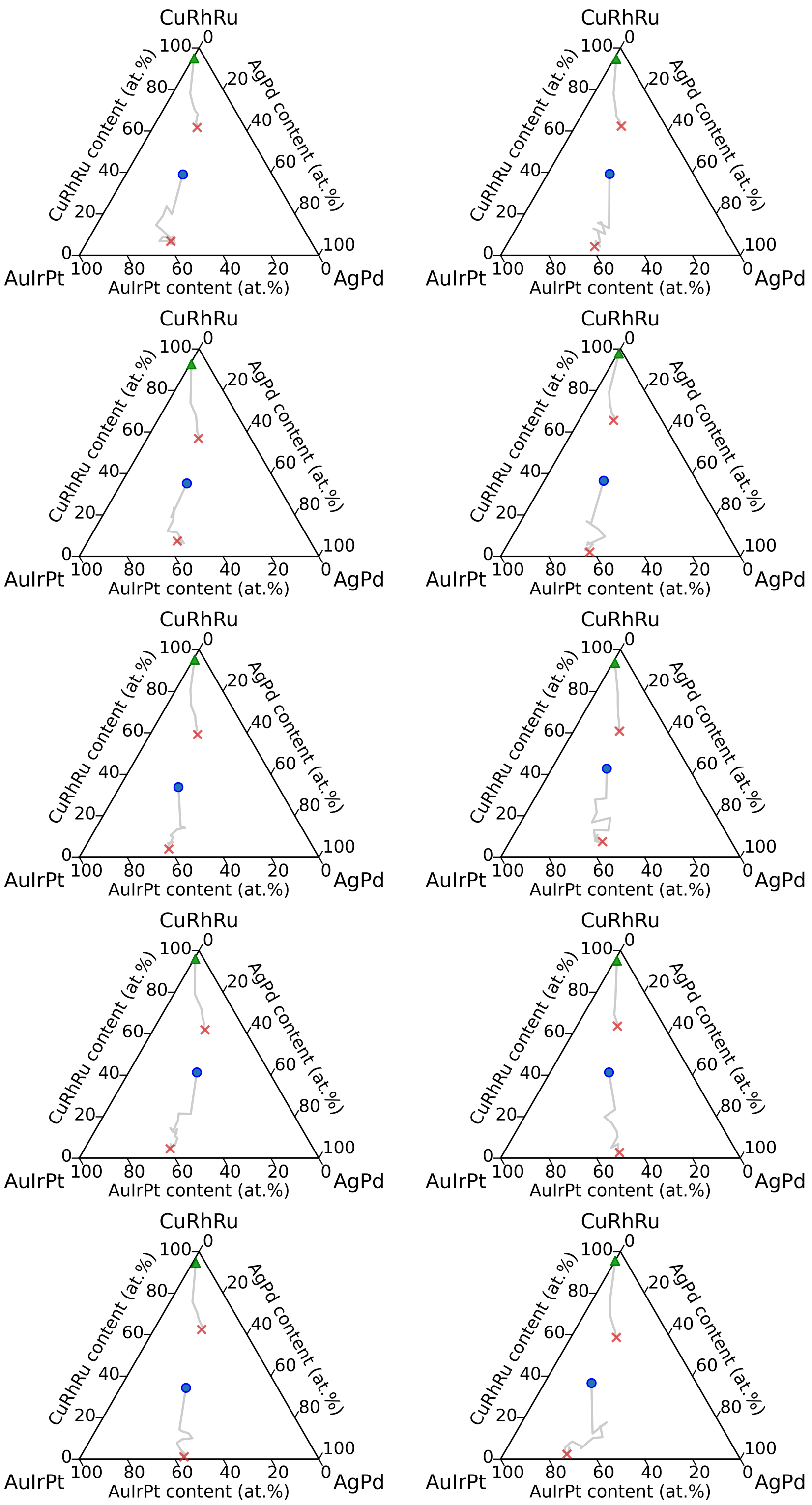

Figure S22: Evolutionary composition paths from Figure 4 shown individually, ordered according to sample numbers in Table S6 from left to right from the top. The paths beginning from the blue circles and green triangles show the (111) surface compositions and total dissolved compositions, respectively.

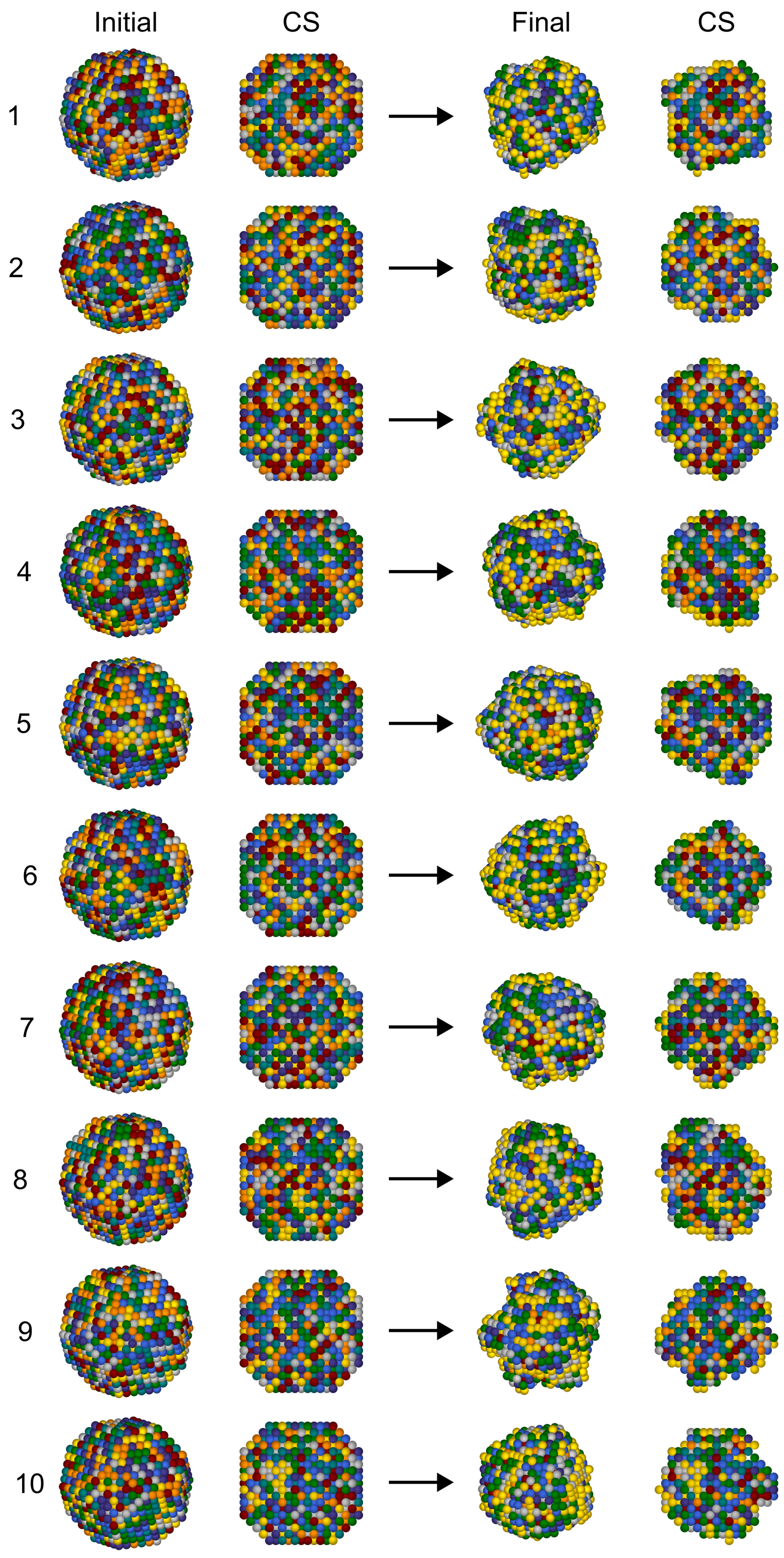

Figure S23: Equimolar particles from Figure 4 and Table S6 before (initial) and after dissolution (final) with cross section (CS).

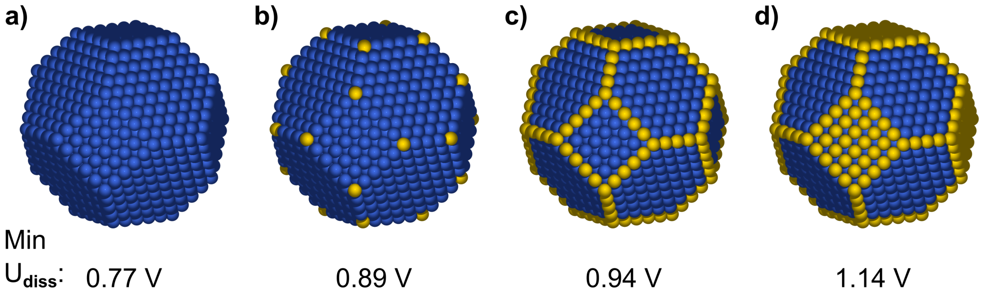

Figure S24: Increase in minimum dissolution potential of Pd nanoparticles through galvanic replacement with Au

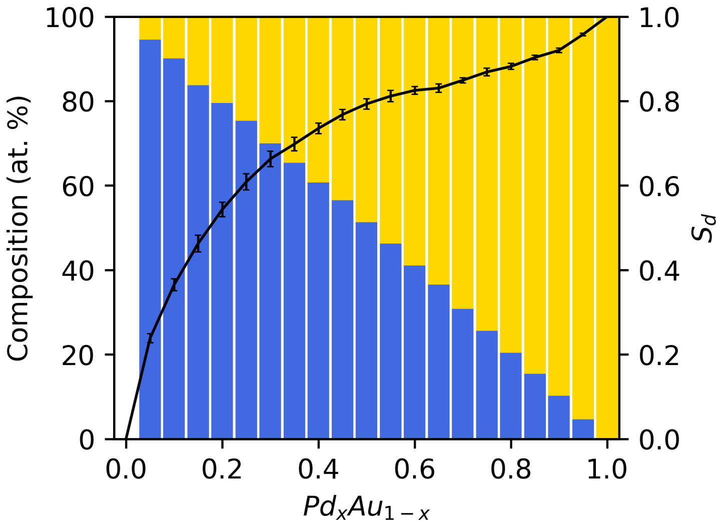

Figure S25: (111) surface composition after dissolution and its stability S_d of $\mathrm{Pd}_x\mathrm{Au}_{1-x}$ alloys

References

[1] J Enkovaara, C Rostgaard, J J Mortensen, J Chen, M Dułak, L Ferrighi, J Gavnholt, C Glinsvad, V Haikola, H A Hansen, and et al. Electronic structure calculations with gpaw: a real-space implementation of the projector augmented-wave method. *Journal of Physics: Condensed Matter*, 22(25):253202, 2010. ISSN 0953-8984. doi: 10.1088/0953-8984/22/25/253202. URL `https://dx.doi.org/10.1088/0953-8984/22/25/253202`.

[2] Ask Hjorth Larsen, Jens Jørgen Mortensen, Jakob Blomqvist, Ivano E Castelli, Rune Christensen, Marcin Dułak, Jesper Friis, Michael N Groves, Bjørk Hammer, Cory Hargus, and et al. The atomic simulation environment—a python library for working with atoms. *Journal of Physics: Condensed Matter*, 29(27):273002, 2017. ISSN 0953-8984. doi: 10.1088/1361-648x/aa680e.

[3] B. Hammer, L. B. Hansen, and J. K. Nørskov. Improved adsorption energetics within density-functional theory using revised perdew-burke-ernzerhof functionals. *Physical Review B*, 59(11): 7413–7421, 1999. ISSN 0163-1829. doi: 10.1103/physrevb.59.7413.

[4] Hendrik J. Monkhorst and James D. Pack. Special points for brillouin-zone integrations. *Phys. Rev. B*, 13:5188–5192, Jun 1976. doi: 10.1103/PhysRevB.13.5188. URL `https://link.aps.org/doi/10.1103/PhysRevB.13.5188`.

[5] Jeffrey Greeley. Structural effects on trends in the deposition and dissolution of metal-supported metal adstructures. *Electrochimica Acta*, 55(20):5545–5550, 2010. ISSN 0013-4686. doi: https://doi.org/10.1016/j.electacta.2010.04.055. URL `https://www.sciencedirect.com/science/article/pii/S0013468610006122`.

[6] Richard Tran, Zihan Xu, Balachandran Radhakrishnan, Donald Winston, Wenhao Sun, Kristin A. Persson, and Shyue Ping Ong. Surface energies of elemental crystals. *Scientific Data*, 3(1):160080, 2016. ISSN 2052-4463. doi: 10.1038/sdata.2016.80. URL `https://dx.doi.org/10.1038/sdata.2016.80`.
